# The role of the cancer stem cell marker CD271 in DNA damage response and drug resistance of melanoma cells

**DOI:** 10.1038/oncsis.2016.88

**Published:** 2017-01-23

**Authors:** T Redmer, I Walz, B Klinger, S Khouja, Y Welte, R Schäfer, C Regenbrecht

**Affiliations:** 1 German Cancer Consortium (DKTK), German Cancer Research Center (DKFZ), Heidelberg, Germany; 2 Laboratory of Molecular Tumor Pathology, Institute of Pathology, Charité—Universitätsmedizin Berlin, Berlin, Germany; 3 Institute for Theoretical Biology, Humboldt-Universität zu Berlin, Berlin, Germany; 4 CPO—Cellular Phenomics and Oncology Berlin-Buch GmbH, Berlin, Germany

## Abstract

Several lines of evidence have suggested that stemness and acquired resistance to targeted inhibitors or chemotherapeutics are mechanistically linked. Here we observed high cell surface and total levels of nerve growth factor receptor/CD271, a marker of melanoma-initiating cells, in sub-populations of chemoresistant cell lines. CD271 expression was increased in drug-sensitive cells but not resistant cells in response to DNA-damaging chemotherapeutics etoposide, fotemustine and cisplatin. Comparative analysis of melanoma cells engineered to stably express CD271 or a targeting short hairpin RNA by expression profiling provided numerous genes regulated in a CD271-dependent manner. In-depth analysis of CD271-responsive genes uncovered the association of CD271 with regulation of DNA repair components. In addition, gene set enrichment analysis revealed enrichment of CD271-responsive genes in drug-resistant cells, among them DNA repair components. Moreover, our comparative screen identified the fibroblast growth factor 13 (FGF13) as a target of CD271, highly expressed in chemoresistant cells. Further we show that levels of CD271 determine drug response. Knock-down of CD271 in fotemustine-resistant cells decreased expression of FGF13 and at least partly restored sensitivity to fotemustine. Together, we demonstrate that expression of CD271 is responsible for genes associated with DNA repair and drug response. Further, we identified 110 CD271-responsive genes predominantly expressed in melanoma metastases, among them were NEK2, TOP2A and RAD51AP1 as potential drivers of melanoma metastasis. In addition, we provide mechanistic insight in the regulation of CD271 in response to drugs. We found that CD271 is potentially regulated by p53 and in turn is needed for a proper p53-dependent response to DNA-damaging drugs. In summary, we provide for the first time insight in a CD271-associated signaling network connecting CD271 with DNA repair, drug response and metastasis.

## Introduction

Despite recent progress in treatment options, malignant melanoma metastasized to liver, lung or brain remains to be a non-curable disease. Overall, the therapy of stage IV melanoma by chemotherapeutics and targeted therapies results in median progression-free survival of approximately 1.5–7 months and a 5-year survival period is observed for 10% of patients only. The major obstacle to long-term patient survival is resistance acquired under therapy. Although BRAF^V600E^ mutated melanomas, which represent 40–60% of this tumor entity, are effectively targetable with the BRAF inhibitor vemurafenib,^1^ relapse occurs as early as within approximately 5 months.^2^ Resistant tumors exhibit upregulation of receptor tyrosine kinase receptors PDGFRB^3^ or EGFR,^4^ signaling mediators such as CRAF or NRAS, as well as mutations in MEK1, MEK2 and NRAS resulting in the stimulation of the RAS/RAF/mitogen-activated protein kinase pathway (reviewed in Spagnolo *et al.*^2^). A recent publication suggested that resistance to vemurafenib was also dependent on the levels of nerve growth factor receptor (NGFR, CD271) and of drug-induced nuclear factor κB (NFκB) signaling.^5, 6^

For therapy of melanomas lacking BRAF or other targetable mutations, the chemotherapeutics paclitaxel, cisplatin and dacarbacine are in clinical use, either administered in combination or as monotherapy.^7^ Dacarbazine, its prodrug temozolomide, as well as fotemustine are favorably used to treat brain metastases because these inhibitors are capable of passing the blood–brain barrier.^8, 9^ Clinical trials combining these drugs with immunological or targeted therapies are ongoing.^10, 11^

It is well known that intrinsic resistance to chemotherapeutics involves the expression of active membrane transporters,^12, 13, 14^ anti-apoptotic genes,^15, 16^ genes associated with stemness^17, 18^ and epigenetic modulators.^17^ The increased DNA repair capacity of melanoma cells^18^ provides an additional mechanism conferring intrinsic resistance to targeted drugs and chemotherapeutics. Although chemotherapy of melanoma cells leads to acquired resistance, DNA-damaging agents induce upregulation of DNA damage-binding protein 2 and xeroderma pigmentosum genes.^19^ It has been shown that acquired resistance of melanoma and glioblastoma to fotemustine is directly linked to high expression of the de-alkylating enzymes.^20^ Thus, acquired increase of the DNA repair capacity is likely to be a major mechanism of melanoma cells in evading chemotherapeutic interventions (reviewed in Soengas and Lowe^21^).

In view of the complexities of resistance mechanisms in chemoresistant melanoma, it is desirable to better understand the interplay of the determining factors and potential ways of their regulation. Therefore, we set out to explore new factors and regulatory mechanisms of therapy resistance by profiling chemoresistant BRAF wild-type melanoma cells. In addition, we analyzed the consequence of drug-induced changes in gene expression in non-resistant cells.

## Results

### Steady-state and induced levels of CD271 protein in resistant and sensitive melanoma cells

**Figure 1:**
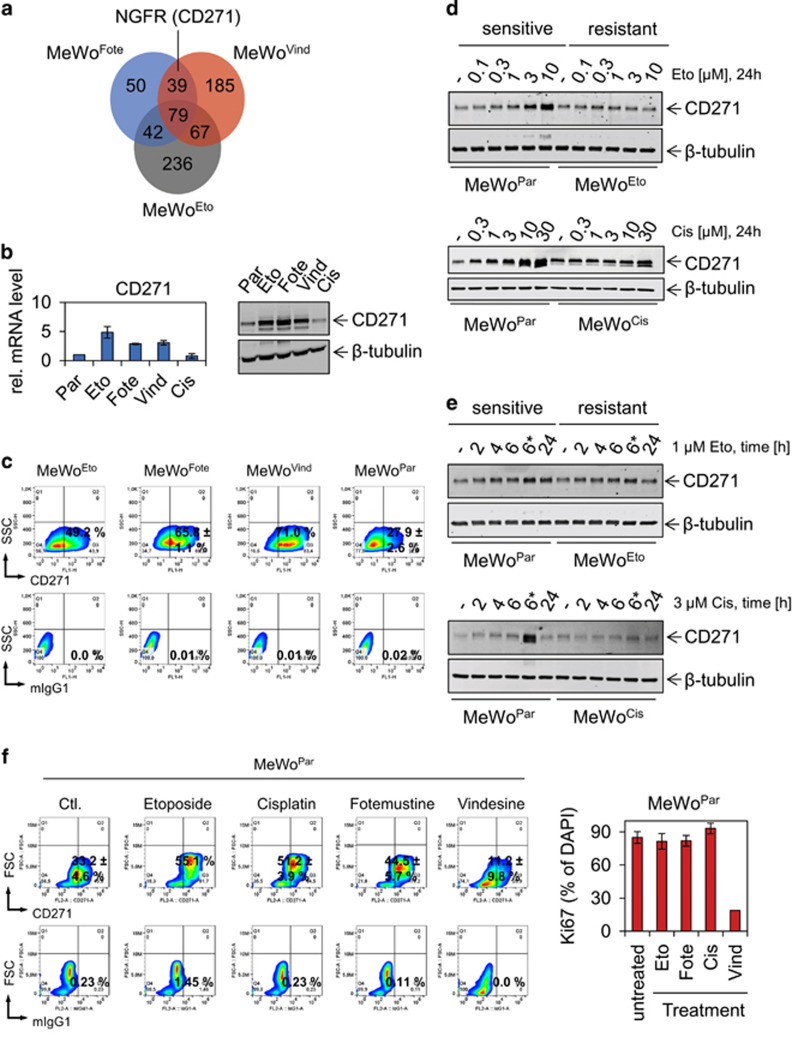
CD271 expression discriminates chemoresistant and sensitive cells. (**a**) Venn diagram depicts unique and commonly top upregulated genes of resistant MeWo cells (fotemustine, MeWo^Fote^, Fote), (vindesine, MeWo^Vind^, Vind) or (etoposide, MeWo^Eto^, Eto) as compared with parental (MeWo^Par^, Par). (**b**) Left panel: verification of CD271 expression in resistant cells by qPCR, shown are relative expression levels±s.d. of independent triplicates. Right panels: immunoblot analysis of cells described in **a**, cisplatin resistant (MeWo^Cis^, Cis), as well as parental cells for levels of CD271. (**c**) Comparative flow cytometry analysis of resistant and parental MeWo cells for levels of CD271. (**d**, **e**) Immunoblot analysis of MeWo^Par^, MeWo^Eto^ and MeWo^Cis^ cells following a dose (etoposide: 0.1–10 μM and cisplatin: 0.3–30 μM both 24 h) and time dependent (2, 4, 6 and 24 h, *etoposide or cisplatin was withdrawn after 6 h, cells were maintained for additional 18 h) treatment for levels of CD271. (**f**) Left panels: analysis of MeWo^Par^ cells following treatment with either etoposide (10 μM), cisplatin (10 μM), fotemustine (30 μg/ml) or vindesine 1 ng/ml for 24 h for cell surface expression of CD271. Right panel: quantification of Ki67-positive MeWo^Par^ cells following drug treatment for 24 h. Shown are numbers of Ki67-positive cells in %±s.d. related to 4′,6-diamidin-2-phenylindol (DAPI). In (**b**, **d**, **e**), whole-cell lysates were analyzed, β-tubulin served as loading control; in (**c**, **f**) 10 000 cells were recorded following incubation with either CD271-PE (top panels) or mouse IgG1 isotype control (mIgG1, lower panels). Shown are mean values±s.d. as indicated.

We performed microarray-based genome-wide expression profiling of the drug-sensitive melanoma cell line MeWo (MeWo^Par^) and of derivatives selected for resistance to etoposide (MeWo^Eto^), fotemustine (MeWo^Fote^), vindesine (MeWo^Vind^) or cisplatin (MeWo^Cis^).^22^ By comparative analysis of profiling data, we identified subsets of genes whose elevated expression was either common or unique to the chemoresistant cells (Figure 1a and Supplementary Tables S1A and B). MeWo^Cis^ cells were exceptional in that there were no marked expression alterations compared with MeWo^Par^ cells. Among the strongly (fold change, FC⩾2, *P*⩽0.05) upregulated factors within the subset of 39 genes common to MeWo^Fote^ and MeWo^Vind^ cells, CD271 (NGFR) attracted our attention because of its propensity to mediate vemurafenib resistance^7^ and stemness in melanoma^23^ (Figure 1a). We verified the elevated transcription of CD271 by quantitative PCR (qPCR) and upregulation at the protein level by immunoblotting (Figure 1b). In addition, we quantified cell surface localization of CD271 by flow cytometry (Figure 1c). On average, we found enrichment of CD271-expressing cells by a factor of 2.23±0.38 in MeWo^Fote^, MeWo^Vind^ and MeWo^Eto^ cells, equivalent to a median proportion of CD271-expressing cells of 62.0±11.4% compared with MeWo^Par^ cells (27.9±2.6%).

Next, we asked whether increased CD271 expression occurred as a direct response to DNA-interfering/DNA-damaging therapeutics or as a secondary effect, nonspecifically induced and maintained during the process of selection for resistant cells. We treated MeWo^Par^ cells with the potent topoisomerase II inhibitor etoposide or the mediator of DNA crosslinks cisplatin in a dosage and time-dependent manner. Etoposide and cisplatin treatment augmented CD271 expression in MeWo^Par^ cells at low and high doses, whereas MeWo^Eto^ or MeWo^Cis^ cells exhibited a constant expression over all concentrations or showed increased CD271 only at highest dose, respectively (Figures 1d and e). Enhanced CD271 protein levels remained elevated for 18 h after etoposide withdrawal (Figure 1e, upper panels). This effect was more evident in the cisplatin time course (Figure 1e, lower panels). We also confirmed upregulation of CD271 cell surface expression following short-term treatment of MeWo^Par^ cells with etoposide and fotemustine but not vindesine by flow cytometry. Treatment with cisplatin showed a very similar effect on CD271 expression (Figure 1f), suggesting transient upregulation at the protein level. Fotemustine treatment of MeWo^Fote^ cells showed no further increase of the CD271 level (Supplementary Figure S1A). The dose-dependent treatment of MeWo^Par^ or MeWo^Vind^ cells showed only marginal or no increase of CD271, respectively (Supplementary Figure S1B). This may suggest a direct response of CD271 to DNA-interfering drugs, acting differently than microtubule-interfering drugs like vindesine. Quantification of Ki67-positive MeWo^Par^ cells revealed no change in response to DNA-interfering drugs (Figure 1f, right panel).

### A CD271-associated gene signature uncovers a regulatory linkage with DNA repair

**Figure 2:**
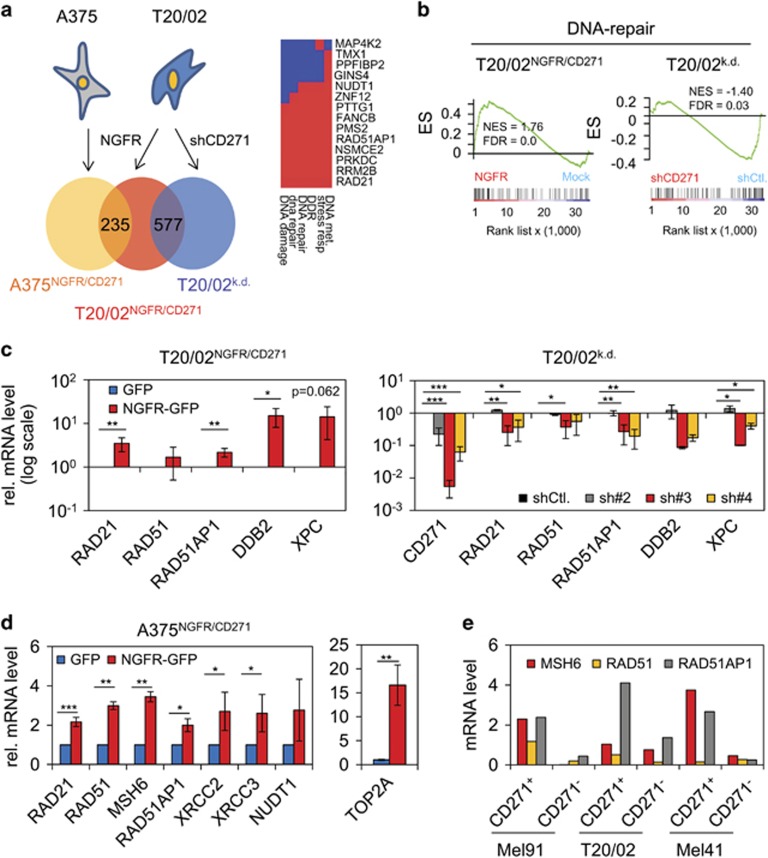
Integrative analysis of melanoma cells with overexpression and/or knock-down of CD271 links CD271 with DNA repair. (**a**) Venn diagram depicting commonly upregulated genes induced in A375 and T20/02 cells engineered to stably express CD271 (A375^NGFR/CD271^, T20/02^NGFR/CD271^) or stably transfected with a CD271-targeting shRNA (shCD271, T20/02^k.d.^). Numbers of identified genes found commonly upregulated in A375^NGFR/CD271^ and T20/02^NGFR/CD271^ (235 genes) or differentially regulated in T20/02^NGFR/CD271^ and T20/02^k.d.^ cells (577 genes, comprising up and downregulated genes) are shown. Right panel: DNA repair cluster from DAVID analysis of a set of 340 CD271-responsive genes upregulated in T20/02 cells, red or blue indicates positive or negative GO-contribution, respectively. (**b**) GSEA of profiling data of T20/02^NGFR/CD271^ and T20/02^k.d.^ cells using a DNA repair signature (230 genes) as provided by MSigDB.^18^ (**c**) Verification of the CD271-dependent regulation of DNA repair genes RAD21, RAD51, RAD51AP1, DNA damage-binding protein 2 (DDB2) and xeroderma pigmentosum genes (XPC) in T20/02^NGFR/CD271^ (left panel) or cells stably transfected with CD271 specific shRNAs (#2, #3, #4) by qPCR (right panel). Expression levels are shown in logarithmic scale, indicating relative expression levels±s.d. of independent triplicates, compared with cells either expressing GFP or knock-down control (shCtl.). *P⩽0.05; **P⩽0.01; ***P⩽0.001. (**d**) CD271-induced expression of DNA repair genes in A375^NGFR/CD271^ cells as determined by qPCR. (**e**) Expression levels of CD271, RAD51AP1, RAD51 and MSH6 in cells of patient-tumor derived cell strains (Mel91, T20/02 and Mel41) sorted for presence or absence of CD271 by fluorescence-assisted cell sorting (FACS). Shown are three independent experiments.

Drug resistance is associated with numerous alterations of gene expression,^24^ hence we asked if CD271 can modulate drug response by impacting on the transcriptome. To identify CD271-responsive transcriptional targets, we performed expression profiling of patient-derived melanoma cells T20/02 and the A375 cell line, both engineered to stably express CD271. Cell lines transfected with the CD271/green fluorescent protein (GFP) transgene showed increased endogenous and ectopic CD271 protein levels and increased cell surface localization (Supplementary Figure S2A). Genome-wide expression profiling revealed 5233 and 736 differentially regulated genes (*P*⩽0.05) in A375^NGFR/CD271^ cells and T20/02^NGFR/CD271^ cells, respectively (Supplementary Figures S2B and C). Of those, 235 genes were upregulated in the two cell lines by CD271 (Figure 2a, Supplementary Table S3). In addition, we contrasted the expression data set of T20/02^NGFR/CD271^ cells with those obtained after silencing endogenous CD271 in the same cell strain.^23^ This comparison identified 577 genes modulated in conjunction with the deregulation of CD271 (Figure 2a). Among those were 340 genes transcriptionally stimulated (⩾1.5-fold) following overexpression of CD271 (*P*⩽0.05) and downregulated (⩽0.5-fold) by CD271 knock-down (*P*⩽0.05), (Supplementary Table S4). In all, 237 genes followed a converse regulation, that is, were repressed by forced CD271 expression (Supplementary Figure S2D, Supplementary Table S5). For a deeper exploration of profiling data, we performed DAVID analysis^25, 26^ of gene subsets and gene set enrichment analysis (GSEA)^27^ of the entire data sets.

DAVID analysis of CD271-induced genes uncovered three clusters associated with gene functions in cell cycle (enrichment score (ES)=1.58), DNA repair (ES=1.34) and cell motility (ES=1.28) (Figure 2a, right panel and Supplementary Figure S3A). To further investigate the association between CD271-responsive genes and DNA repair, we performed GSEA, particularly considering a DNA repair gene signature deposited in Broad Institute's molecular signature database (MSigDB, http://software.broadinstitute.org/gsea/msigdb/index.jsp). Indeed, GSEA revealed enrichment of genes involved in DNA repair (nominal enrichment score=1.76) in CD271-overexpressing cells (T20/02^NGFR/CD271^) and conversely regulated in CD271 knock-down cells (T20/02^k.d.^) (Figure 2b). We confirmed the differential, CD271-dependent expression of RAD21, RAD51 (homologs of *S. pombe* and *S. cervisiae*, respectively), RAD51-associated protein 1 (RAD51AP1) genes, responsible for double-strand break repair, and of DNA damage-binding protein 2 and xeroderma pigmentosum genes involved in nucleotide excision repair (Wiese *et al.*^28^ and reviewed in Sancar *et al.*^29^) (Figure 2c). In A375^NGFR/CD271^ cells, RAD21, RAD51AP1 and other repair genes including MSH6 (homolog of *Escherichia coli* MutS) were equally upregulated in a CD271-dependent manner (Figure 2d and Supplementary Figure S3C). To further prove the relationship between CD271 and increased expression of repair genes, we sorted three different patient-derived melanoma cell strains for high endogenous and low CD271 expression by fluorescence-assisted cell sorting. The expression pattern of the repair genes RAD21, RAD51, RAD51AP1 and MSH6 was correlated with that of CD271 in positively and negatively sorted cells (Figure 2e and Supplementary Figure S2E).

### CD271 regulates expression of FGF13, a mediator of chemoresistance

**Figure 3:**
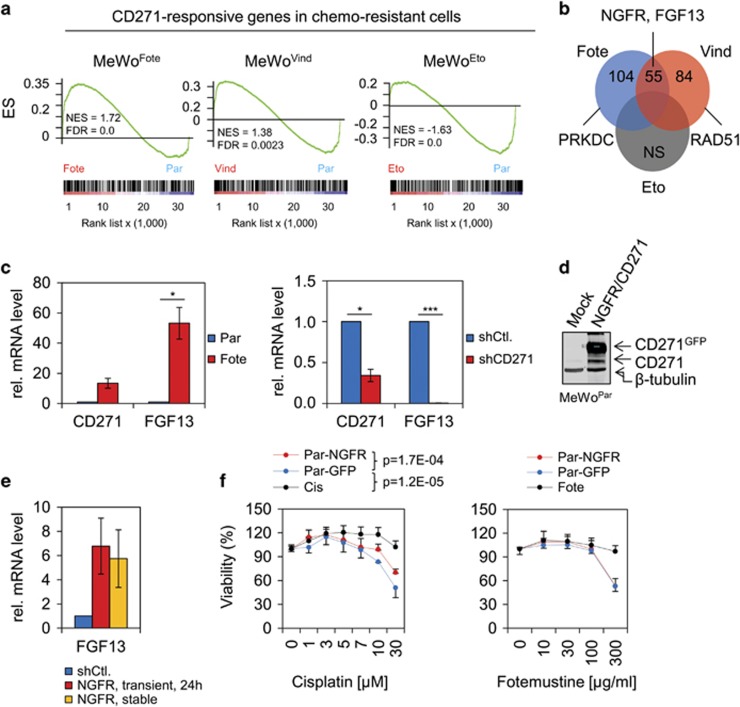
CD271-responsive genes are predominantly expressed in chemoresistant cells. (**a**) GSEA with profiling data of MeWo^Fote^, MeWo^Vind^ and MeWo^Eto^ cells and a consensus signature, comprising 516 CD271-responsive genes. FDR, false discovery rate; NES, nominal enrichment score; NS, no significant enrichment. (**b**) Venn diagram depicting commonly found CD271-responsive genes in chemoresistant cells. FGF13 represents the most common gene in MeWo^Fote^ and MeWo^Vind^ cells. (**c**) Left panel: qPCR of MeWo^Fote^ and MeWo^Par^ cells and right panel: MeWo^Fote^ cells stably transfected with a validated CD271-targeting shRNA (shCD271) or control (shCtl.) for expression of CD271 and FGF13. (**d**) Immunoblot analysis of whole-cell lysates of MeWo^Par^ cells either non-transfected (Mock) or engineered to stably express CD271 (NGFR, GFP-tagged) for levels of CD271. (**e**) Expression level of FGF13 in MeWo^Par^ cells either stably or transiently expressing CD271. (**f**) Determination of viability of MeWo^Cis^ (Cis) and MeWo^Fote^ (Fote) cells as compared with stably GFP (Par-GFP) or NGFR/CD271-expressing MeWo^Par^ (Par-NGFR) cells following a dose-dependent treatment with either cisplatin (μM) or fotemustine (μg/ml). Viability is indicated in %±s.d. of *n*=8 technical replicates. A representative out of *n*=3 biological replicates is shown.

To further exploit the relationship between CD271 expression and chemoresistance, we performed a supervised analysis of the expression profiles of MeWo^Fote^, MeWo^Vind^, MeWo^Eto^ and MeWo^Par^ cells by GSEA based on a consensus set of 516 upregulated CD271-responsive genes identified in T20/02^NGFR/CD271^ and A375^NGFR/CD271^ cells (Supplementary Table S6). Compared with MeWo^Par^ cells, we observed enrichment of CD271-responsive genes most evident in MeWo^Fote^ and MeWo^Vind^, but not MeWo^Eto^ cells (Figure 3a). We further asked for specific enrichment of genes involved in DNA repair and cell cycle in chemoresistant cells by using of GSEA and a subset of genes of the CD271 consensus signature, constituting both processes. We found highest enrichment of CD271-responsive genes associated with DNA damage and cell cycle processes in MeWo^Fote^ and MeWo^Vind^ but not MeWo^Eto^ cells (Supplementary Figure S4A). Genes of this subset followed a CD271-dependent regulation as validated in engineered cells (Supplementary Figure S4A, lower panels).

In addition, GSEA led to identification of a CD271-responsive gene signature in MeWo^Fote^ and MeWo^Vind^ cells (Figure 3b), comprising important components of the DNA damage repair machinery such as PRKDC (DNA-dependent protein kinase catalytic subunit) and RAD51 and the cisplatin resistance associated gene encoding fibroblast growth factor 13 (FGF13).^30^ We confirmed high expression of FGF13 in MeWo^Fote^ cells by qPCR (Figure 3c) and found a remarkable (250-fold) downregulation upon silencing of CD271 in those cells (MeWo^Fote-shCD271^) (Figure 3c, right panel). In addition, MeWo^Par^ engineered cells to overexpress CD271 (MeWo^Par-NGFR/CD271^) showed increased levels of FGF13 mRNA, suggesting a CD271-dependent expression of FGF13 (Figures 3d and e). Finally, we asked whether forced expression of CD271 in MeWo^Par^ cells renders them refractory to cisplatin. We observed that overexpression of CD271 (NGFR) indeed significantly decreased the sensitivity of parental MeWo cells to cisplatin but not to fotemustine, etoposide or vindesine (Figure 3f and Supplementary Figures S4B and D) as compared with GFP-expressing (MeWo^Par-GFP^) or non-transfected (Par) control cells.

### Knock-down of CD271 partially restores drug response in fotemustine-resistant cells

**Figure 4:**
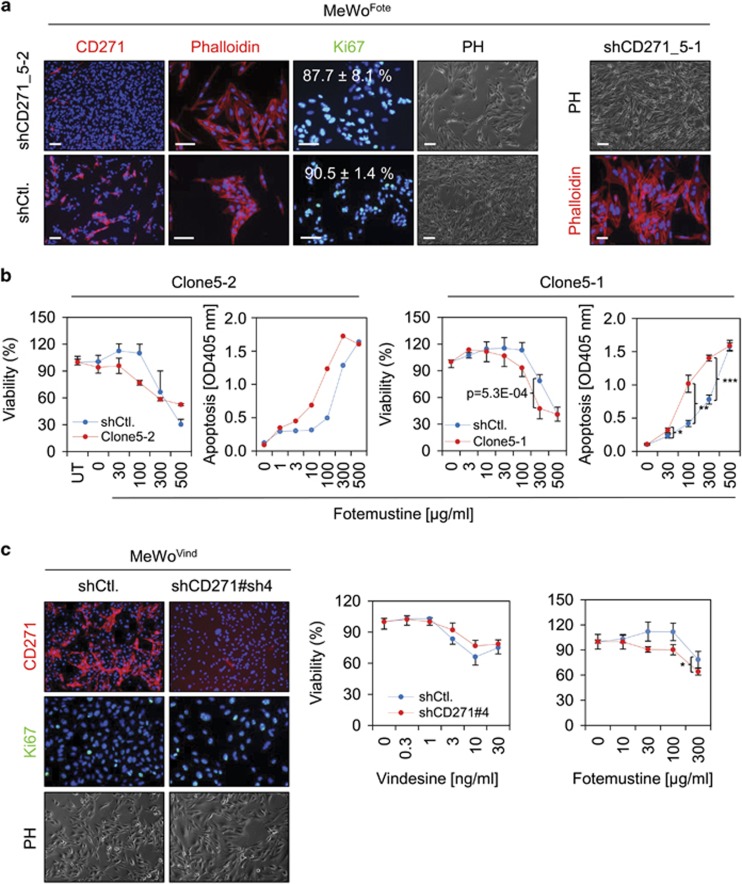
Levels of CD271 expression modulate drug response. (**a**) Left panels: IF microscopy of MeWo^Fote^ cells stably transfected with a validated CD271-targeting shRNA (shCD271_5-2, clone 5-2) or control (shCtl.) for CD271 (red, left panels), phalloidin (red, center panels), presence of Ki67 (green, right panels) or bright field is shown. Numbers represent % of Ki67-positive cells±s.d. of *n*=4 counts related to 4′,6-diamidin-2-phenylindol (DAPI) that served as nuclear stain, scale bars indicate 50 μm. Right panel: IF microscopy of clone 5-1 (shCD271_5-1) for phalloidin or bright field is shown. (**b**) Left and right panels: determination of cell viability and apoptosis of MeWo^Fote^ cells, clone 5-2/clone 5-1 modified as described in **a** following a dose-dependent treatment with fotemustine (μg/ml) for 48 h. Viability is indicated in %±s.d. of *n*=8 replicates, related to untreated (100% viability) cells, a representative out of three biological replicates is shown, UT=untreated; 0=solvent control. Apoptosis was determined by an ELISA-based measurement of apoptosis induced DNA-fragmentation of clone 5-2 and clone 5-1 after 48 h, scales indicate apoptosis as OD405 values, *P⩽0.05; **P⩽0.01; ***P⩽0.001. (**c**) Left panels: IF microscopy of MeWo^Vind^ cells following stable knock-down of CD271 by a validated shRNA (sh#4) for levels of CD271 (red), Ki67 (green) or bright field. Center and right panel: determination of cell viability CD271 knock-down cells (pool) following a dose-dependent treatment with vindensine and fotemustine for 48 h. Representative experiments out of *n*=3 are shown.

To investigate a causal role of CD271 in chemoresistance, we stably knocked-down CD271 expression in MeWo^Fote^ cells (MeWo^Fote-shCD271^, Figure 3c right panel and Supplementary Figure S5A). CD271 silencing induced moderate morphological changes of cells grown in monolayers but no significant inhibition of proliferation as determined by counting of Ki67-positive cells (Figure 4a). Next, we assessed the susceptibility of CD271 knock-down cells to fotemustine. In MeWo^Fote-shCD271^ cells (clones shCD271_5-2 and shCD271_5-1), the susceptibility to fotemustine was partially restored as indicated by reduced viability of clone 5-2 at 100 μg/ml (76.9±2.6% vs 109.9±10.1%) and clone 5-1 at 300 μg/ml (47.5±11.5% vs 78.6±7.1%), as well as by enhanced apoptosis following 48-h treatment (Figure 4b). In addition, we explored the response of clone 5-1 to cisplatin, vindesine and etoposide and observed that none of these showed a comparable response as compared with fotemustine (Supplementary Figure S5B). Moreover, we performed a stable knock-down of CD271 in MeWo^Vind^ cells, by two independent short hairpin RNAs (shRNAs; sh#3, sh#4) yielding in a robust downregulation of CD271 (Figure 4c and Supplementary Figure S5C, left panel). However, knock-down of CD271 did not sensitize cells to vindesine, etoposide or cisplatin (Figure 4c, Supplementary Figure S5C and data not shown) but at least partly increased the response to fotemustine (Figure 4c, right panel).

### CD271-responsive genes confer a metastatic phenotype on melanoma cells

**Figure 5:**
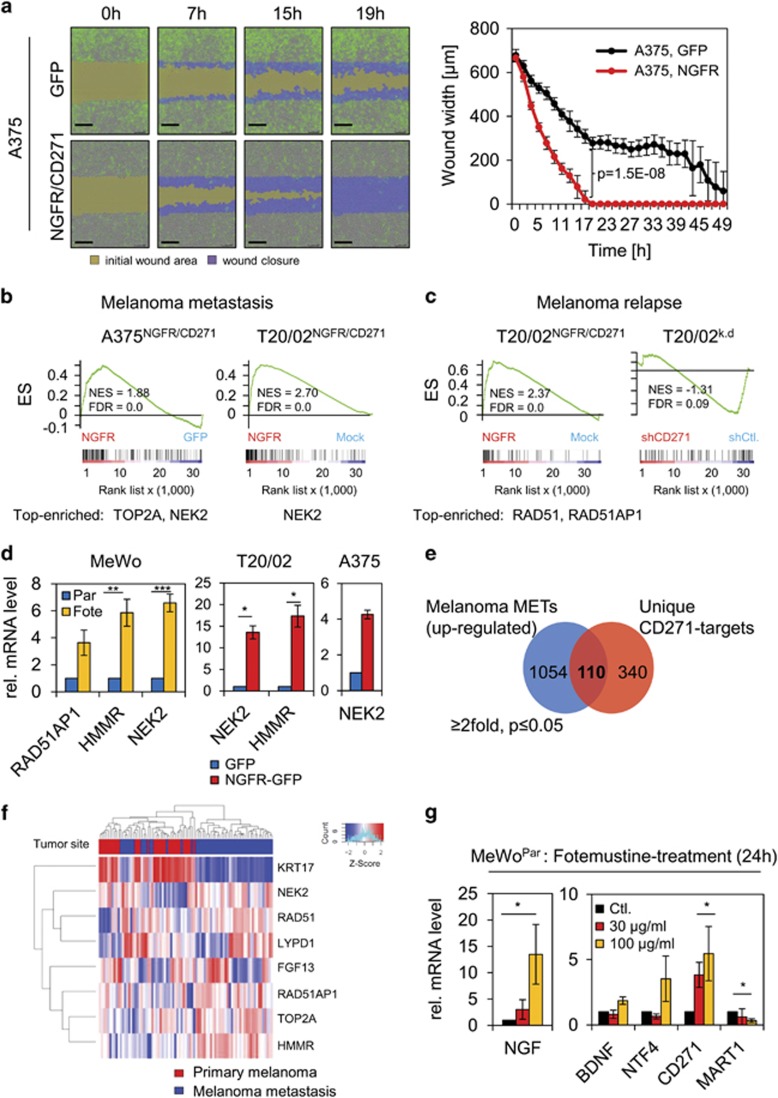
Expression of CD271-responsive genes facilitates discrimination of primary tumor and melanoma metastases. (**a**) Left panels: migratory capacity of A375 cells stably transfected with either GFP or CD271 (NGFR/CD271, GFP positive) at time points 0, 7, 15 and 19 h after wounding. Wound closure is indicated by an increasing migration front (blue) and a decreasing wound area (yellow). Right panel: quantification of wound closure. Shown are average wound widths (μm) of *n*=8 replicates relative to time of imaging (h) of GFP (black) or NGFR (red) transfected cells. Scale bars indicate 200 μm. (**b**, **c**) GSEA of profiling data of A375^NGFR/CD271^ or T20/02^NGFR/CD271^ or T20/02^k.d.^ cells and signatures indicating melanoma metastasis^46^ or melanoma relapse.^18^ Top enriched genes are indicated. (**d**) Expression levels of potential mediators of drug resistance NEK2, HMMR and RAD51AP1 in MeWo^Fote^ cells as compared with MeWo^Par^. Center and right panel: validation of a CD271-dependent expression of NEK2 and HMMR or NEK2 in T20/02^NGFR/CD271^ or A375^NGFR/CD271^ cells, respectively. (**e**) Venn diagram depicting overlapping genes found among data sets of melanoma metastasis (1054 genes) of study GSE8401 as provided by the GEO database and CD271-responsive genes identified in T20/02 cells (340 genes). (**f**) Supervised clustering of selected CD271-regulated genes depicting distribution in a set of primary tumors and melanoma metastasis. Tumor site is color coded. (**g**) Expression levels of known ligands potentially binding to CD271, for example, NGF, brain-derived neurotrophic factor (BDNF) and neurotrophin 4 (NTF4) in MeWo^Par^ cells following treated with fotemustine (30, 100 μg/ml) for 24 h (left and center panels) as determined by qPCR. In (**d**, **g**), scales indicate relative expression levels±s.d. of independent triplicates, *P⩽0.05; **P⩽0.01; ***P⩽0.001.

As selective pressure occurring upon drug-based therapies can lead to changes in expression levels of genes favoring metastasis and relapse,^31, 32^ we asked whether expression of CD271 and its responsive genes may be involved in mediating a migratory/metastatic phenotype. We took advantage of a life cell imaging system based wound-healing assay, which allowed us to monitor migration of A375^NGFR/CD271^ and MeWo^Par-NGFR/CD271^ and control cells (A375^GFP^, MeWo^Par-GFP^) within 2 days. We observed that overexpression of CD271 in A375 and MeWo cells specifically increased the migratory phenotype and accelerated wound closure in both cellular systems (Figure 5a, Supplementary Figure S6A and Supplementary Movie Files 1–4). In addition, we observed that MeWo^Vind^ and MeWo^Fote^ cells showed a 3.9-fold and 1.2-fold increased migration as compared with MeWo^Par^ cells (Supplementary Figures S6B and C and Supplementary Movie Files 5 and 6).

Using GSEA, we recovered the metastasis and relapse gene signature of Broad Institutes' MSigDB within the data sets of A375^NGFR/CD271^ and T20/02^NGFR/CD271^ cells (Figures 5b and c and Supplementary Figure S7A). The CD271-responsive genes RAD51AP1 and the known resistance mediators NIMA-related kinase 2 (NEK2)^33^ and Hyaluronan-mediated motility receptor (HMMR),^34^ as well as topoisomerase II were highly expressed in MeWo^Fote^ cells (Figure 5d, left panel and data not shown). The CD271-dependent upregulation of NEK2, HMMR and TOP2A observed by GSEA was validated (Figure 5d, center and right panel and Figure 2d). To further assess whether expression of CD271-responsive genes may not only promote chemoresistance but also metastasis formation, we took advantage of publicly available expression profiling data of primary (*n*=31) and metastatic melanoma (*n*=52).^35^ Exploration of the set of upregulated genes (*n*=1054, >2-fold, *P*⩽0.05) revealed the presence of 110 CD271-responsive genes, upregulated in melanoma metastases (Figure 5e and Supplementary Table S7).

Among them, we found NEK2, RAD51AP1 and LYPD1 (LY6/PLAUR domain containing 1), a neuronal transmembrane protein potentially associated with brain metastases.^36^ Statistical analysis revealed that expression of the CD271-responsive genes NEK2 (*P*=1.56E–06), RAD51AP1 (*P*=2.67E–04), HMMR (*P*=5.47E–07) and TOP2A (*P*=1.56E–06) but not FGF13 (*P*=0.3122) significantly discriminated primary tumors and melanoma metastases (Figure 5f and Supplementary Figures S7B and C). A predominant expression of RAD51AP1 was in addition observed in a small set of brain metastases (Supplementary Figure S7D).^37^ Keratin 17 was found inversely correlated with expression of CD271-responsive genes in melanoma metastases (Figure 5f), upregulated upon CD271 knock-down (data not shown). The role of CD271 as mediator of melanoma metastasis is further underpinned by the inverse regulation of the known suppressor of melanoma metastasis KISS1^38^ and DMBT1 (deleted in brain tumors 1). DMBT1 expression is frequently lost in lung cancer^39^ and glioblastoma.^40^ We observed a strong upregulation of both genes upon knock-down of CD271 in MeWo derivatives and T20/02 cells (Supplementary Figures S6D and E), suggesting that CD271 expression leads to repression of both metastasis inhibitors, facilitating melanoma metastasis.

As we identified mediators of melanoma metastasis among CD271-responsive genes, we next asked whether these genes can be induced in MeWo^Par^ cells by fotemustine treatment. We observed increased expression of NEK2 and RAD51AP1 (Supplementary Figure S7E, left panel) as well as strong increased levels of the ligands of CD271, nerve growth factor (NGF, ~13.5-fold) and neurotrophin 4 (~3.5-fold) (Figure 5g) following fotemustine treatment for 24 h. Brain-derived neurotrophic factor was only marginally increased, melanoma antigen recognized by T cells 1 showed inverse correlation with CD271 and neurotrophins. In conclusion, the enhanced expression of neurotrophins like NGF upon drug treatment and in drug-resistant cells (MeWo^Fote^) (Supplementary Figure S7E, right panel) suggests that the genetic programs governing drug resistance and metastasis may overlap at least partially.

### CD271 is induced in a p53-dependent manner

To assess the mechanisms regulating the drug-mediated induction of CD271, we explored signaling processes potentially induced on drug treatment of MeWo^Par^ cells. Pathway components included stabilized p53 (p-p53^S15^), p21^CIP^ (CDKN1A), mitogen-activated protein kinase (extracellular signal-regulated kinases 1 and 2 (ERK1/2), p38 kinase), AKT signaling as well as checkpoint-kinase 1 (CHK1), a direct target of ataxia telangiectasia mutated and ataxia telangiectasia and RAD3-related signaling. The latter is the DNA damage sensor pathway and marks sites of DNA damage via formation of γH2AX a crucial step initiating DNA repair.^29^ We also determined phosphorylation of IκBα and the nuclear localization of NFκB/p65, as activation of NFκB signaling was earlier recognized as a proposed mechanism of CD271 regulation.^7^

**Figure 6:**
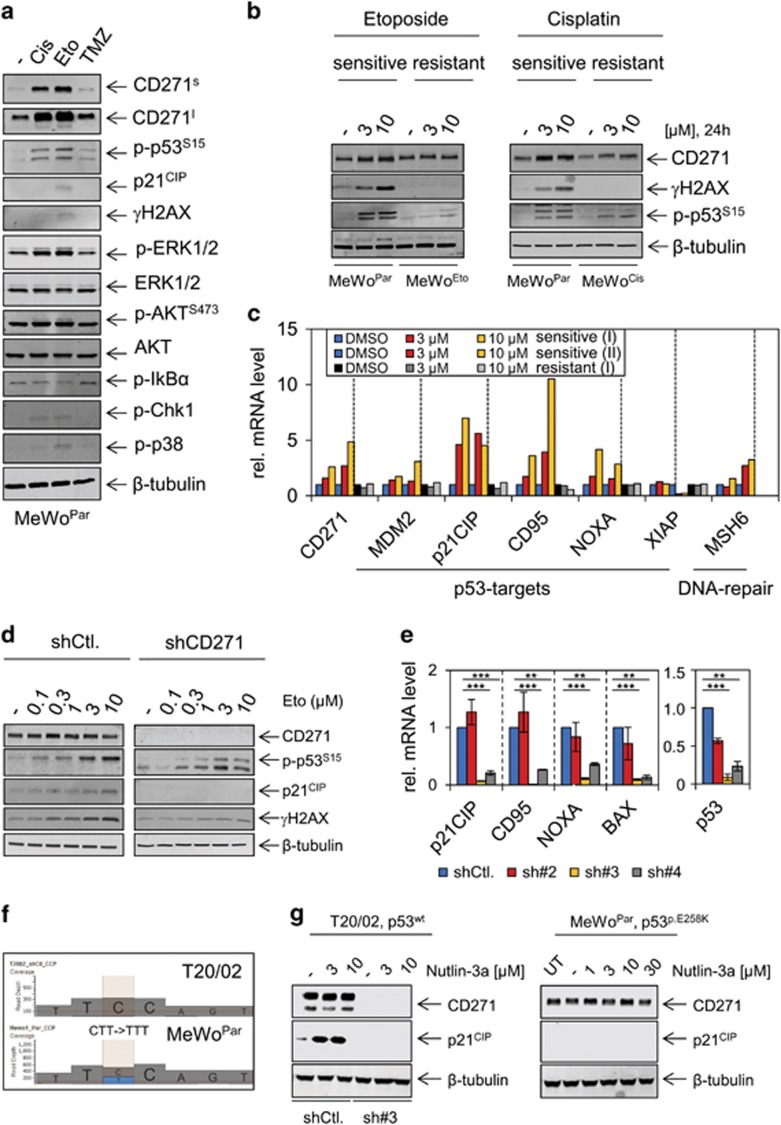
Mechanistic insights in the regulation of CD271. (**a**) Immunoblot analysis of MeWo^Par^ cells treated with either cisplatin (Cis), etoposide (Eto) or temozolomide (TMZ) (all at a final concentration of 10 μM) for levels of CD271 (s/l=short/long exposure), p-p53^S15^, p21^CIP^, γH2AX, p-ERK1/2 and ERK1/2, p-AKT^S473^ and AKT, p-IκBα, p-CHK1 and p-p38 kinase. (**b**) Immunoblot analysis of MeWo^Eto^, MeWo^Cis^ or MeWo^Par^ cells following dose-dependent treatment with etoposide or cisplatin (3, 10 μM both) for 24 h, respectively. Levels of CD271, γH2AX and phosphorylated (activated) p53 (p-p53^S15^) are shown indicating the different response of sensitive and resistant cells to drugs. (**c**) Regulation of p53-targets MDM2, p21^CIP^ (CDKN1A), CD95, NOXA, XIAP, as well as MSH6 and CD271 in MeWo^Par^ and MeWo^Eto^ cells treated with etoposide (3, 10 μM) for 24 h. Shown are relative expression levels of representative experiments (I, II). (**d**) Immunoblot analysis of T20/02 cells stably transfected with shCD271 or control, following dose-dependent treatment with etoposide for levels of CD271, p-p53^S15^, p21^CIP^ and γH2AX. (**e**) Analysis of cells described in **d** but stably transfected with different CD271-targeting shRNAs (sh#2, sh#3, sh#4) or control (shCtl.) for levels of p53, p21^CIP^, CD95, NOXA and BAX. Scale indicates relative expression levels±s.d. of independent triplicates, *P⩽0.05; **P⩽0.01; ***P⩽0.001. In (**a**, **b**, **d**), whole-cell lysates were analyzed, β-tubulin served as loading control. (**f**) Panel-based next-generation sequencing (NGS) revealed detection of a TP53 mutation (p.E258K, 5′-GAA->AAA-3' antisense (3′-CTT→TTT-5′)) in MeWo^Par^ cells, not present in wild-type T20/02 cells. Shown are sequences, as well as read depths. (**g**) Left panel: immunoblot analysis of cells described in **d** for levels of CD271 and p21^CIP^ following a dose-dependent treatment with MDM2 inhibitor nutlin-3a. Right panel: nutlin-3a treatment of MeWo^Par^ cells.

We observed a strong activation of mitogen-activated protein kinase signaling (p-ERK1/2) and the p53-signaling pathway following treatment with cisplatin (Cis), etoposide (Eto) or temozolomide, as well as a moderate activation of p38 kinase and CHK1 (Figure 6a), however, we did not detect activation of canonical NFκB signaling (Figure 6a and Supplementary Figure S8A). In contrast to MeWo^Par^ cells, p53 was not stabilized in chemoresistant MeWo^Eto^ and MeWo^Cis^ cells upon drug treatment (Figure 6b). Moreover, known p53 targets^41^ and CD271 were co-regulated in etoposide-treated MeWo^Par^ but not in MeWo^Eto^ cells (Figure 6c), supporting the conclusion that the p53 pathway is impaired in resistant cells. We observed a lower induction of p53 targets upon treatment with cisplatin in MeWo^Par^ cells as compared with etoposide and also observed a weak regulation of targets in MeWo^Cis^ cells (Supplementary Figure S8B). To further assess the connection between p53 and CD271, we investigated the response of the p53 pathway in a patient-derived cell strain following stable knock-down of CD271 and dose-dependent treatment with etoposide. Etoposide treatment increased levels of γH2AX, p-p53^S15^ and p21^CIP^ in control cells but not in CD271 knock-down cells (Figure 6d). The levels of p53 itself, of p53 targets, as well as of the DNA damage sensors PRKDC, Nijmegen breakage syndrome 1 and ataxia telangiectasia mutated were downregulated in CD271 knock-down cells. Expression of ataxia telangiectasia and RAD3-related was not affected (Figure 6e and Supplementary Figure S9A). In line with downregulation of DNA repair genes, knock-down cells showed increased DNA damage (Supplementary Figure S9A, center and right panels). These data suggest that endogenous levels of CD271 are required for proper drug response. We observed comparable CD271-dependent regulations of p53 targets and sensors of DNA damage repair in MeWo^Fote^ and MeWo^Vind^ cells upon knock-down of CD271 albeit with lower significance (Supplementary Figures S9A and B).

The co-regulation of p53 and CD271 in drug-exposed MeWo^Par^ cells may suggest that CD271 upregulation is controlled by p53. The CD271 promoter harbors three potential binding sites matching the consensus sequence of p53 as predicted by p53FamTaG algorithm (http://p53famtag.ba.itb.cnr.it/) (Supplementary Figure S10A). To elucidate, if p53 indeed controls CD271 expression, we treated T20/02 and MeWo^Par^ cells with the MDM2 inhibitor nutlin-3a, which stabilizes p53 leading to activation of downstream signaling, in p53 wild-type cells. Panel-based next-generation sequencing of MeWo^Par^, resistant derivatives and T20/02 cells revealed the presence of a TP53 p.E258K mutation in MeWo^Par^ and resistant cell lines and evidenced the wild-type status of T20/02 cells (Figure 6e, Supplementary Figure S10B and Supplementary Table S2A). In line with this observation, we observed a strong increase in p21^CIP^ but not CD271 in T20/02 (shCtl.) but not CD271 knock-down (sh#3) or MeWo^Par^ cells (Figure 6f). These data suggest that knock-down of CD271 impairs activation of p53-dependent signaling processes by inhibition of MDM2 as well as DNA-damaging drugs. In addition, qPCR and immunofluorescence (IF) microscopy showed comparably low levels of mutant p53 in all MeWo derivatives (Supplementary Figures S10C and D). Further, next-generation sequencing and copy number analysis revealed the presence of a mutation (p.I2018F) or amplification of PRKDC in MeWo^Cis^ or MeWo^Fote^ cells (Supplementary Figure S10E), respectively. Mutations in additional genes involved in DNA repair, for example, Fanconi Anemia Complementation Group A (FANCA) found in MeWo^Fote^ cells or amplification of Meiotic Recombination 11 Homolog A (MRE11A) observed in MeWo^Vind^ cells (Supplementary Figure S10E) may render cells susceptible to drugs by increased genomic instability.

## Discussion

Stem cell-like properties are considered as a major cause for rendering cancer cells intrinsically resistant to therapeutics.^42, 43^ NGFR (tumor necrosis factor superfamily member 16 or CD271) has been used as a marker to enrich for tumor-initiating cells in melanoma.^44^ Our own previous findings had indicated that CD271 not only regulates stem cell functions, for example, tumorigenicity and plasticity, but was also highly expressed in patient-derived cell cultures from melanoma metastases.^23^ These results prompted the current investigation as to whether CD271 was involved in chemoresistance, particularly in wild-type BRAF melanomas not eligible for anti-BRAF kinase therapy. We show here that selection of MeWo cells for acquired resistance to chemotherapeutics triggering DNA damage via alkylation, cross-linking of DNA or topoisomerase inhibition was accompanied by high CD271 expression. High expression of CD271 was maintained in resistant cells, with exception of MeWo^Cis^ cells, which may have developed a different mechanism to overcome cisplatin-induced DNA damage without the need for CD271. However, short-term exposure of drug-sensitive cells to chemotherapeutics induced CD271 expression already after 4 h, suggesting that CD271 upregulation and drug exposure were directly linked rather than due to selection of highly expressing cells present in a heterogeneous cell population. Moreover, silencing of CD271 partially re-sensitized resistant cells toward drug treatment and identified a link between enhanced CD271 expression and the regulation of FGF13, a known mediator of cisplatin resistance.^30^

Upregulation of CD271 in response to drugs occurred in a p53-dependent manner. However, the relationship between CD271 and p53 appeared to be more complex and additional general mechanisms involved in stress response^45^ may trigger CD271 expression. In drug-sensitive melanoma cells, in which intrinsically moderate CD271 expression was silenced by introduction of shRNA, the p53-dependent DNA repair system was impaired because of low expression or activation of p53 and of crucial proteins acting as DNA damage sensors PRKDC, Nijmegen breakage syndrome 1 and ataxia telangiectasia mutated. This suggested that a sufficient level of CD271 in turn is required for the proper response of cells to chemotherapeutic drugs. This conclusion is based on the results of gene expression profiling of cells in which CD271 was either silenced or enhanced by ectopic expression of a recombinant CD271 expression vector. CD271-related gene signatures comprised predominantly DNA repair genes, cell cycle progression genes and genes involved in metastasis. This may explain why DNA repair genes are expressed at a higher level in CD271-expressing chemoresistant melanoma cells in the absence of p53 expression levels exceeding those in drug-sensitive cells. However, a CD271-independent upregulation of the DNA repair machinery cannot be excluded at this stage.

Moreover, although our survey points out a role of CD271 in drug resistance, we only observed a partial sensitization of MeWo^Fote^ or MeWo^Vind^ cells to certain drugs. In addition, overexpression of CD271 was unable to fully mimic the resistant phenotype of observed in any of the resistant cells lines. This suggests that additional mechanisms are present in chemoresistant melanoma cells and clonal heterogeneity supposedly lead to variabilities in drug response.

**Figure 7:**
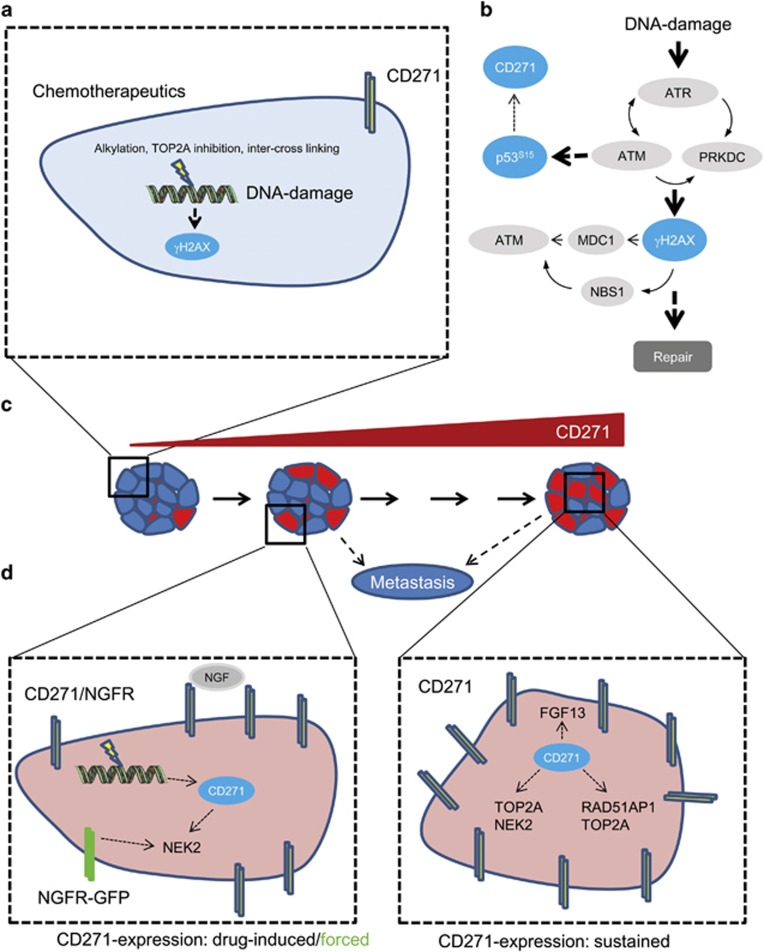
Proposed model for the CD271 regulation and potential downstream effects mediating drug resistance and metastasis. (**a**) Alkylating drugs (fotemustine, TMZ), TOP2A inhibitors (etoposide) or inter-cross-linking agents (cisplatin) induce expression of CD271 in MeWo^Par^ cells. (**b**) Drug-dependent DNA damage results in activation of the ataxia telangiectasia mutated (ATM)/ataxia telangiectasia and RAD3-related (ATR) pathway leading to formation of stabilized p53 (p-p53^S15^) and γH2AX. Stabilized p53 may regulate expression of CD271. (**c**) Phenotypic consequences associated with increased CD271 expression levels. Left: drug-sensitive cell population (CD271 low), middle: Short-term drug treatment of sensitive cell population (CD271 intermediate), right: drug-resistant cell population (CD271 high), prone to metastasis. (**d**) Left: Downstream consequences of enhanced CD271/NGFR expression by drug treatment or overexpression (NGFR-GFP). Right: sustained expression of CD271 regulating drug resistance (FGF13, TOP2A, NEK2) and metastasis-associated (RAD51AP1, TOP2A) genes.

Besides DNA repair genes, ectopic CD271 expression impinged on target genes, which have previously been associated with melanoma relapse and metastasis.^18, 46^ Our survey of potential CD271-associated genes in publicly available melanoma expression profiles recovered as many as 110 CD271-responsive genes predominantly expressed in metastases, among them the NEK2 gene (Never in mitosis gene A-related kinase 2), previously found associated with drug resistance in myeloma^33^ and RAD51AP1 previously associated with metastasis of melanoma and breast cancer^18, 47^ as well as TOP2A (topoisomerase II). Expression of TOP2A was associated with worse prognosis in melanoma.^48^ NEK2 was also identified as a regulator of aldehyde dehydrogenase 1, a marker of cancer-initiating cells,^49^ again linking stem cell function and drug resistance. A hypothetical model depicting the regulation of CD271 and downstream effects is shown in Figures 7a and d.

Metastasis to the brain is frequently observed in melanoma patients, therapeutically treated with drugs able to pass the blood–brain barrier, such as fotemustine, temozolomide or dacarbacine. Recent publications demonstrated that expression of CD271 is frequently observed in melanoma brain metastases.^50^ These findings are in line with our observation defining a crucial role of CD271 in regulating metastasis gene expression and conferring chemoresistance. Moreover, CD271 expression was found in brain metastatic melanoma cells resistant to vemurafenib.^8^ These observations underpin our finding that CD271-responsive genes were also predominantly expressed in a small set of matched melanoma brain metastases. Notably, NGF mediates melanoma cell migration and metastasis as reported previously.^51^

High expression of metastasis-associated CD271-responsive genes NEK2, RAD51AP1, TOP2A and NGF in drug-resistant MeWo cells and their drug-dependent regulation, may have consequences for the priming of melanoma cells for metastasis. Although FGF13 shows no statistical significant discrimination between melanoma primary tumors and metastases, we observed that subsets of melanoma metastases show high expression of either FGF13 or LYPD1. The role of both genes are poorly understood in melanoma metastases, however, FGF13, which is a special type of FGFs, is not only associated with signaling processes but also has a role in microtubule stabilization and the regulation of neuronal polarization and migration.^52^ Hence, FGF13 may present a new driver of melanoma metastasis.

In general, these findings lead to the provocative hypothesis that sustained expression of CD271 may activate a genetic program promoting simultaneously drug resistance and metastasis. CD271-expressing cells potentially start to metastasize earlier than CD271-negative cells. This is further underpinned by a very recent work^53^ and our observation that melanoma cells with a high endogenous level or overexpression of CD271 showed increased migration into a scratch wound, suggesting CD271 as a determining factor in melanoma metastasis and a candidate factor determining drug response.

## Materials and methods

### Cell culture

All primary low passage melanoma cell strains, as well as their derivatives were cultured in Quantum 263 tumor growth medium (Q236, Capricorn Scientific, Marburg, Germany) supplemented with 1% penicillin/streptomycin (Invitrogen, Carlsbad, CA, USA). The patient tumor-derived cell strain T20/02 has been established from a lymph node metastasis. A375 cells (ATCC, Manassas, VA, USA) and MeWo cell lines were cultured in Leibovitz's L-15 medium (Invitrogen) supplemented with 10% fetal bovine serum, 1%, penicillin/streptomycin (Invitrogen), transferrin/fetuin, minimum essential medium-vitamins, insulin, glucose and sodium bicarbonate. Next-generation sequencing of T20/02 and MeWo cells revealed absence of mutations in BRAF or RAS (K/H/N), Supplementary Table S2A. Cells were cultured in 5% CO_2_, 37 °C and routinely passaged at 80% confluence. Medium was changed every 3 days. Stably shRNA or control transfected fotemustine-resistant cells were selected for puromycine resistance (10 μg/ml) and sub-cloned by limited dilution assays, which yielded in clones 5-1 and 5-2. Vindesine resistant cells were transfected with shRNA or control plasmids, selected for generation of cell pools.

### Drug treatment assays

Drug treatments were performed for at least 48 h in six-well plates (2.5 × 10^5^ cells seeded) for RNA/protein extraction or 96-well plates (2500 cells per well seeded) for viability assays. Stock solutions of etoposide (10 mM, dimethylsulfoxide), cisplatin (1670 mM, sodium chloride, 0.9%), temozolomide (10 mM, dimethylsulfoxide) and nutlin-3a (10 mM, dimethylsulfoxide), (all from Sigma-Aldrich, Munich, Germany) and fotemustine (50 mg/ml, ethanol, 80%) were used. Fotemustine was received either directly from the hospital and administered to cells at day of preparation, or received from company (Sigma-Aldrich). Viability was determined by MTT assay using 50 μl of MTT solution (5 mg/ml) added 48 h after drug treatment. Concentration of dimethylsulfoxide-solubilized formazan was detected at 570 nm after 1 h of incubation.

### Flow cytometry/fluorescence-assisted cell sorting

Fluorescence-assisted cell sorting and flow cytometry were performed as reported previously.^23^ Data analysis was done with FlowJo software (http://www.flowjo.com/).

### Immunoblot

Total cell lysates were prepared in sodium dodecyl sulfate lysis buffer (1% sodium dodecyl sulfate, 10 mM Tris-HCl, 2 mM EDTA). In all, 10–25 μg of whole-cell lysates were separated on 12% sodium dodecyl sulfate–polyacrylamide gel electrophoresis gels and transferred on a nitrocellulose membrane by using the iBlot Dry Blotting System (Invitrogen). Membranes were blocked with Odyssey Blocking Buffer/phosphate-buffered saline-Tween (0.1%), (1:1, LI-COR Biosciences, Lincoln, NE, USA) for 1 h. Incubation with primary antibodies (p75NTR clone D4B3XP recognizing the total CD271, p-ERK1/2, ERK1/2, p-AKT(S473), AKT, γH2AX, p21CIP, p-p53(S15), p-IĸBα, p-p38, p-Chk1 and β-tubulin, clone 9F3; all 1:1000; from Cell Signaling Technology, New England Biolabs GmbH, Frankfurt am Main, Germany) was done overnight at 4 °C. Incubation with secondary antibodies donkey anti-rabbit immunoglobulin G or donkey anti-mouse both (diluted 1:10 000; LI-COR Biosciences) was done for 1 h at room temperature. Signals were detected with the Odyssey Infrared Imaging System (LI-COR Biosciences).

### RNA isolation and quantitative reverse transcriptase–PCR

RNA isolation was performed as reported previously.^23^ Primers were designed using the PrimerQuest Tool (http://eu.idtdna.com/primerquest/home/index) for 55–60 °C annealing temperatures and product size of 100–250 bp (Supplementary Table S8).

### Immunofluorescence

IF was performed as reported previously.^23^ Incubation with primary antibodies CD271-PE (Miltenyi, Bergisch Gladbach, Germany, clone ME20.4-1.H4, mouse IgG1, 1:100), γH2AX (1:250, Cell Signaling Technology), NFκB/p65 and p53 (Cell Signaling Technology, 1:100) or TRITC-labeled phalloidin (1:1000, Sigma-Aldrich) diluted in blocking buffer was done overnight at 4 °C. Second day, cells were washed 3x with phosphate-buffered saline, and incubated with secondary antibodies AlexaFluor488/555 or 594 (1:500) recognizing either rabbit or mouse-produced antibodies and 4′,6-diamidin-2-phenylindol (Sigma-Aldrich, 1:500) for 1 h at room temperature. Washed cells were covered with 500 μl phosphate-buffered saline and used for microscopy. IF pictures were recorded with Zeiss Axiovert40CFL with accompanied Illuminator HPX120C and software AxioVision Rel. 4.8 (all Carl Zeiss AG, Oberkochen, Germany).

### Live cell imaging-based cell migration assay

Migration of model cell lines overexpressing CD271 (A375^NGFR/CD271^; MeWo^Par-NGFR/CD271^) or controls (A375^GFP^; MeWo^Par-GFP^), as well as fotemustine or vindesine resistant cells (MeWo^Fote^; MeWo^Vind^) was performed with the scratch wound cell migration assay (Essen Bioscience Ltd, Welwyn Garden City, Hertfordshire, UK) following the manufacturer's instructions. Briefly, 30 000 cells of each cell line were seeded 24 h before, yielding in a dense cell layer. Reproducibly wounds were scratched with the Wound Maker, a block with 96 pins. After wounding, the 96-well plate was placed into the Incucyte ZOOM life-cell imaging system (Essen Bioscience Ltd.). Cell migration was monitored every 2 h for 2 days, using a × 10 magnification. Serial pictures were stacked for movie preparation using the ImageJ software (https://imagej.nih.gov/ij/). Statistical analysis was performed by using a two-tailed, paired *t*-test.

### Generation of stable cell lines

Knock-down of CD271 was performed as reported previously.^23^ For overexpression, melanoma cells were stably transfected with a plasmid expressing GFP-tagged human NGFR (RG207966, OriGene, Rockville, MD, USA) and selected with G418 (100–300 μg/ml, PAA). Stable GFP-expressing control cell lines were established by viral transduction with pLenti-PGK-GFP (Addgene, Cambridge, MA, USA). Briefly, 5 × 10^6^ 293LTV (BIOCAT GmbH, Heidelberg, Germany) were transfected with 4.5 μg pLenti-PGK-GFP plasmid and 4.5 μg packaging plasmid (pLenti Combo Mix I, HIV Lentiviral Packaging Plasmid Mix, BIOCAT) using calcium chloride precipitation. Virus containing supernatant was collected 48 h after transfection and concentrated (~50-fold) using PEG-it solution (SBI, Palo Alto, CA, USA).

### Gene expression profiling

Whole-genome expression profiling of chemoresistant and sensitive melanoma cells (MeWo, GSE78898) or cells of a patient-derived cell strain (T20/02) stably transfected either with a CD271-targeting (T20/02^k.d.^) or a control shRNA (GSE52456) plasmid, as well as T20/02 and A375 cells stably expressing either NGFR/CD271 or GFP (GSE78155) was performed with three biological replicates. Gene expression of parental (*n*=1) and chemoresistant derivatives (Eto, Fote *n*=3; Vind, Cis *n*=2) was measured on two Illumina BeadChips (HumanHT-12V4, Illumina Inc., San Diego, CA, USA). After summarization via BeadStudio, data on each chip was quantile normalized (R package preprocessCore) and filtered (detection *P*<0.01 for at least one sample). After combination of chips by quantile normalization and batch correction (R package sva), significance thresholds for differential regulation were set to mean FC⩾2 and adjusted *P*-value <0.05 (R package limma). Plots and heat maps were generated using R packages ggplot2 and heatmap.3 with spearman's rank correlation as similarity measure and complete linkage.

### Gene set enrichment analysis

GSEA was performed with appropriate software (GSEA version 2.2.2, Cambridge, MA, USA), as well as signatures specific for DNA repair, melanoma relapse and melanoma metastasis were received from the latest version of the Molecular Signatures Database v5.1 (MSigDB, GSEA) of the Broad Institute (http://software.broadinstitute.org/gsea/downloads.jsp). The CD271-responsive gene signature represents a consensus (*n*=516 genes) of genes upregulated in A375^NGFR/CD271^ and/or T20/02^NGFR/CD271^ cells. DAVID analysis was used to derive a sub-signature (*n*=187 genes) from the consensus signature comprising genes associated with DNA repair and cell cycle processes.

### Meta-analysis

Exploration for presence of CD271-responsive genes, publicly available data were used comprising primary melanoma with melanoma metastases of different organs (GSE8401) or extracranial with brain metastases (GSE50496). From the latter study, only matched pairs of extracranial and brain metastases were taken into consideration for analysis. Data were downloaded from GEO (http://www.ncbi.nlm.nih.gov/geo). Statistical analysis of CD271-responsive genes differentially expressed among melanoma metastases and primary melanoma was done in R using a modified version of the linear model function (lm).

### Panel-based sequencing and copy number variation (CNV)

Panel-based next-generation sequencing was performed following the manufacturer's instructions. Briefly, 10 ng of genomic DNA were used for preparation of barcoded PCR-libraries using the comprehensive cancer panel. Libraries (100 pM of each primer pool per cell line) were mixed, amplified and bead-coupled by emulsion PCR using the OneTouch 2 system. Sequencing of duplexed samples was performed on the Ion Personal Genome Machine (PGM) System and a 318v2 chip (all equipment from Thermo Fisher Scientific, Waltham, MA, USA). Variant call files were analyzed by our in-house pipeline and mutations were selected regarding prediction of potential impact on protein function by algorithms SIFT/PolyPhen^54, 55^ and MutationTaster.^56^ Analysis of CNV was performed with R package CNVPanelizer using BAM/BAI sequencing output files. Determination of CNVs of resistant MeWo cells was referenced to sensitive (MeWo^Par^) cells.

### Statistics

All quantitative real-time reverse transcriptase–PCR data are presented as mean±s.d. values. Where indicated, Student's *t*-test (two-tailed) was used to compare two groups. A *P*-value ⩽0.05 is considered statistically significant. Center values shown represent median values. Data sets subjected to pairwise comparisons performed using Student's *t*-test were checked for normality and variance among groups via calculating Pearson's coefficients fell within a range of ±0.8. For statistics of microarray data, see section Gene expression profiling.
