# Supplementary material for: The role of the cancer stem cell marker CD271 in DNA damage response and drug resistance of melanoma cells
Source: Oncogenesis. 2017 Jan 23;6(1):e291–. doi: 10.1038/oncsis.2016.88 (PMC5294251; doi:10.1038/oncsis.2016.88)
Supplement: Supplementary Information [file oncsis201688x1.docx]

**Supplementary Figures and Tables**

**Figure S1: Drug response of MeWo cells. (A)** Flow cytometry of MeWo^Fote^ cells following treatment with fotemustine (30 µg/ml) for 24h. Shown are mean values ± SD as indicated. **(B)** Immunoblot analysis of MeWo^Par^ and MeWo^Vind^ cells for levels of CD271 following a dose-dependent treatment with vindesine for 24 h.

**Figure S2: Expression of CD271 is linked with DNA-repair. (A)** Left panels: immunofluorescence microscopy of stable (A375, Mel91) or transient (T20/02) NGFR/CD271 transfected cells for expression of CD271 (red). DAPI served as nuclear stain, scale bars indicate 50 µm. Right panels: immunoblot of engineered cells depicting total levels of both endogenous (CD271) and exogenous (CD271-GFP). **(B-C)** Volcano plots of expression profiling data of T20/02^NGFR/CD271^ or A375^NGFR/CD271^ cells. Depicted are up- and down-regulated genes as fold increase related to either Mock or GFP transduced cells, shown in log10 or -log10 (p-value). Red dotted line marks the threshold of p ≤ 0.05. **(D)** Schematic representation of unique CD271-responsive genes identified in T20/02 cells with either overexpression or knock-down of CD271 as indicated. **(E)** Absolute (ΔCT) levels of CD271 and RAD21 in sorted cells.

**Figure S3: Gene-ontology (GO) analysis uncovers CD271-associated cellular processes. (A-B)** GO analysis for genes induced (DNA-repair and cell cycle associated genes, red) or repressed (angiogenesis associated genes, blue) by expression of CD271. Enrichment of genes in % is shown. **(C)** GSEA with expression profiling data of A375^NGFR/CD271^ cells and a signature representing genes associated with DNA-repair.

**Figure S4: CD271-responsive genes are enriched in drug-resistant cells. (A)** Top panels: enrichment of CD271-responsive genes associated with DNA-repair and cell cycle (n=187) in MeWo^Fote^ and MeWo^Vind^ but not MeWo^Eto^ cells as determined by GSEA is shown. Bottom panels: validation of the CD271-dependent regulation of genes associated with DNA-repair and cell cycle progression, provided by a specific signature used in (A). Enrichment of genes is shown by overexpression of CD271 (A375^NGFR/CD271^, T20/02^NGFR/CD271^) and depletion by knock-down of CD271 (T20/02^k.d.^). **(B)** Determination of cell viability of MeWo^Par^ cells either stably transfected with GFP (Par-GFP) or NGFR/CD271 (Par-NGFR) or non-transfected (Par-Mock) following a dose-dependent treatment with cisplatin. **(C)** Viability of Par-GFP or Par-NGFR cells following dose-dependent a treatment with etoposide or vindesine. **(D)** Viability of Par-NGFR, MeWo^Par^ (Par) and MeWo^Fote^ (Fote) cells following a dose-dependent treatment with fotemustine.

**Figure S5: CD271 knock-down in MeWo^Vind^ cells only marginally affects drug response. (A)** Immunoblot of MeWo^Fote^ cells, stably transfected with shCtl., or a CD271-targeting shRNA (sh#3) showing a robust knock-down of CD271 in independent clones5-2 and 5-1. **(B)** Determination of cell viability of clone5-1 following a dose-dependent treatment with cisplatin, vindesine and etoposide for 48 h. Shown are representative experiments. **(C)** Left panel: immunoblot of MeWo^Vind^ cells, stably transfected with shCtl., or CD271-targeting shRNAs (sh#3, sh#4) showing a robust knock-down of CD271. Right panel: viability of MeWo^Vind^ cells with a stable knock-down of CD271 (shCD271#3) following a dose-dependent treatment with vindesine.

**Figure S6: Expression of CD271 determines the migratory phenotype of melanoma cells. (A-C)** Measurement of the migratory capacity of MeWo^Par^ cells stably transfected with GFP (MeWo^Par-GFP^) or NGFR/CD271 (MeWo^Par-NGFR^) or MeWo^Fote^ and MeWo^Vind^ cells as indicated by images and declining wound widths. The dashed line indicates the lowest wound width determined with MeWo^Par-NGFR^ cells for comparison with MeWo^Vind^ cells. Shown are median values ± SD of n=8 replicates for each time-point. **(D)** qPCR for levels of metastasis inhibitors KISS1 and DMBT1 in MeWo^Vind^, MeWo^Par^, MeWo^Fote^ and T20/02 cells with stable knock-down of CD271. Shown are relative expression levels ± SD of independent triplicates, scale is logarithmic. **(E)** p-values indicating significance of gene regulation shown in (D).

**Figure S7: CD271 drives expression of metastasis-related genes in melanoma. (A)** Enrichment of melanoma relapse associated genes in A375^NGFR/CD271^ cells as determined by GSEA. **(B-C)** Box plots depicting expression levels of 5 CD271-responsive genes in primary melanoma (red) and melanoma metastases (blue). **(D)** Venn-diagram depicts analysis of matched pairs of brain and extracranial metastases (lymph node, soft tissue) of three patients selected from study GSE50496 for overlap with CD271-responsive genes identified in melanoma metastases (GDS3966, skin, lymph node). Numbers indicate CD271-responsive genes found enriched in brain metastases of the respective matched pair. Commonly found genes are indicated. **(E)** Left panel: determination of levels of RAD51AP1 and NEK2 in MeWo^Par^ cells, treated with fotemustine (100 µg/ml) for 24 h (left panel) and right panel: expression of NGF in MeWo^Fote^ cells as compared to MeWo^Par^.

**Figure S8: CD271 knock-down decreases levels of DNA-damage sensors ATM, PRKDC and NBS1. (A)** Immunofluorescence microscopy of MeWo^Par^ cells treated with fotemustine (100 µg/ml) and etoposide (10 µM) for 24 h or non-treated are shown. Localization of NFkB/p65 and levels of γH2AX and CD271 are shown. DAPI served as nuclear stain, scale bars indicate 50 µm. Arrows indicate rare cells with nuclear NFkB/p65. **(B)** Regulation of p53-targets MDM2, p21^CIP^ (CDKN1A), CD95, NOXA and CD271 in MeWo^Par^ and MeWo^Cis^ cells treated with cisplatin (3, 10 µM) for 24 h. Shown are relative expression levels ± SD.

**Figure S9: Regulation of DNA-damage sensors by CD271. (A) Left panel:** levels of sensors of DNA-damage PRKDC, NBS1, ATR and ATM in T20/02 cells following shRNA mediated knock-down of CD271. Scale indicates relative expression levels ± SD of independent triplicates, *p≤0.05; **p≤0.01; ***p≤0.001. Center and right panels: immunofluorescence microscopy of CD271 knock-down (sh#3) and control (shCtl.) cells (T20/02) for levels of γH2AX; quantification of γH2AX positive cells related to DAPI. **(B)** Left and center panels: regulation of p53-targets and sensors of DNA-damage in clones5-1 and 5-2 (MeWo^Fote^) as well as in MeWo^Vind^ cells with a stable knock-down of CD271 (cell pool, right panel) as determined by qPCR. Shown are relative expression levels ± SD.

**Figure S10: Expression of mutated p53 is low and maintained in drug-resistant cells. (A)** Consensus sequence of a p53-binding site as determined by p53FamTaG. **(B)** Mutation status of DNA-repair genes MLH1, MSH6, FANCA, PRKDC and TP53 of MeWo cells as determined by panel-based NGS. **(C)** qPCR of cell lines analyzed in (A) shows relative and total levels of p53. HPRT served as reference gene. **(D)** Immunofluorescence microscopy of MeWo^Par^ cells as well as resistant cells MeWo^Fote^, MeWo^Eto^ and MeWo^Cis^ for total levels of p53. **(E)** Copy number analysis (CNV) of relevant DNA-repair genes of MeWo^Vind^, MeWo^Eto^, MeWo^Cis^ and MeWo^Fote^ cells related to MeWo^Par^. Color coded changes of some DNA-repair genes unique for a certain cell line, are indicated.

**Supplementary Tables**

**Table S1A: Expression profiling of MeWo cells.** Shown are differentially regulated genes (p≤0.05) of, MeWo^Vind^, MeWo^Fote^, MeWo^Eto^ and MeWo^Cis^ cells as fold change (FC) compared to parental MeWo^Par^ cells.

**Table S1B: Expression profiling of MeWo cells.** Shown are significantly up- or down-regulated genes (p≤0.05) of MeWo^Vind^ (S1B_1/4), MeWo^Fote^ (S1B_2/5), and MeWo^Eto^ (S1B_3/6) cells as fold change (FC) compared to parental MeWo^Par^ cells.

**Table S2A: Summary of damaging/effective mutations as determined in MeWo cells by panel-based NGS.** Shown are mutations of genes with a damaging impact as predicted by SIFT/PolyPhen or MutationTaster.

**Table S2B: CNV analysis of MeWo cells.** Copy number changes relative to MeWo^Par^ cells are shown. Considering a comparable aneuploidy of MeWo^Par^, MeWo^Vind^, MeWo^Fote^, MeWo^Eto^ and MeWo^Cis^ cells, CNV are related to MeWo^Par^ cells. CNVs were determined via CNVPanelizer.

**Table S3: CD271-responsive genes up-regulated in T20/02^NGFR/CD271^ and A375^NGFR/CD271^ cells.** The table shows 235 genes found up-regulated in T20/02^NGFR/CD271^ and A375^NGFR/CD271^ cells comprising genes associated with melanoma metastasis, DNA-repair, cell cycle, p53-signaling or drug-resistance.

**Table S4: CD271-responsive genes identified in T20/02 cells (p≤0.05), induced.** The list summarizes 340 genes most significantly up-regulated in a CD271-dependent manner. These genes were found down-regulated or up-regulated by knock-down or overexpression of CD271, respectively.

**Table S5: CD271-responsive genes identified in T20/02 cells (p≤0.05), repressed.** The list summarizes 237 genes most significantly regulated in a CD271-dependent manner. These genes were found up-regulated or down-regulated by knock-down or overexpression of CD271, respectively.

**Table S6: A consensus set of CD271-responsive genes.** The list summarizes 516 consensus genes found up-regulated in a CD271-dependent manner in T20/02^NGFR/CD271^ and/ or A375^NGFR/CD271^ cells.

**Table S7: CD271-responsive genes present in melanoma metastases.** The list summarizes 110 CD271-responsive genes found predominantly expressed in melanoma metastases as compared to primary melanoma.

**Table S8: qPCR primer sequences.** The table summarizes sequences of primers used for qPCR-based validation of gene expression levels as observed by genome-wide expression profiling.
