# Supplementary figures and images for: The role of the cancer stem cell marker CD271 in DNA damage response and drug resistance of melanoma cells
Source: Oncogenesis. 2017 Jan 23;6(1):e291–. doi: 10.1038/oncsis.2016.88 (PMC5294251; doi:10.1038/oncsis.2016.88)

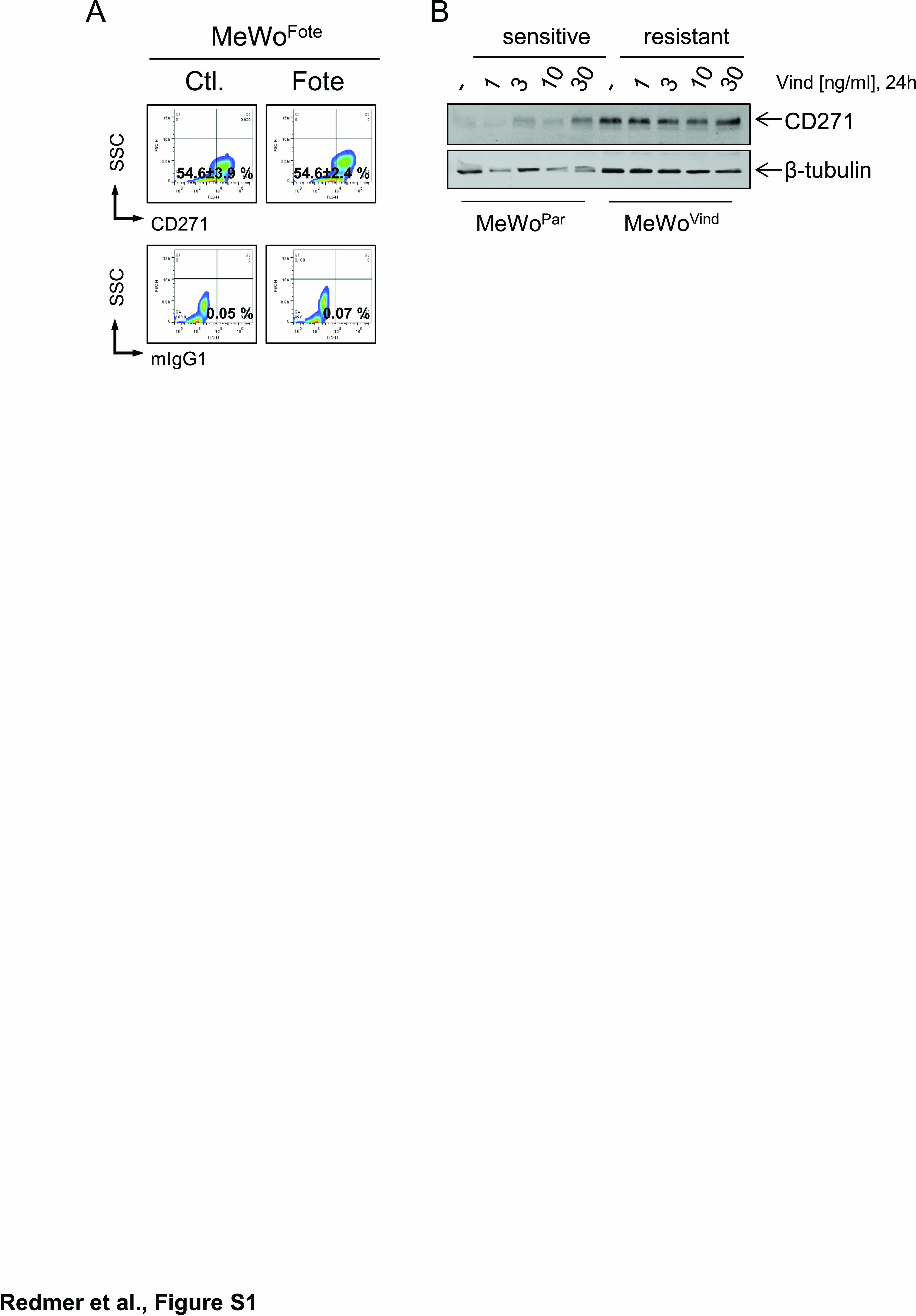

Supplement: Supplementary Figure 1 [file oncsis201688x8.tif]

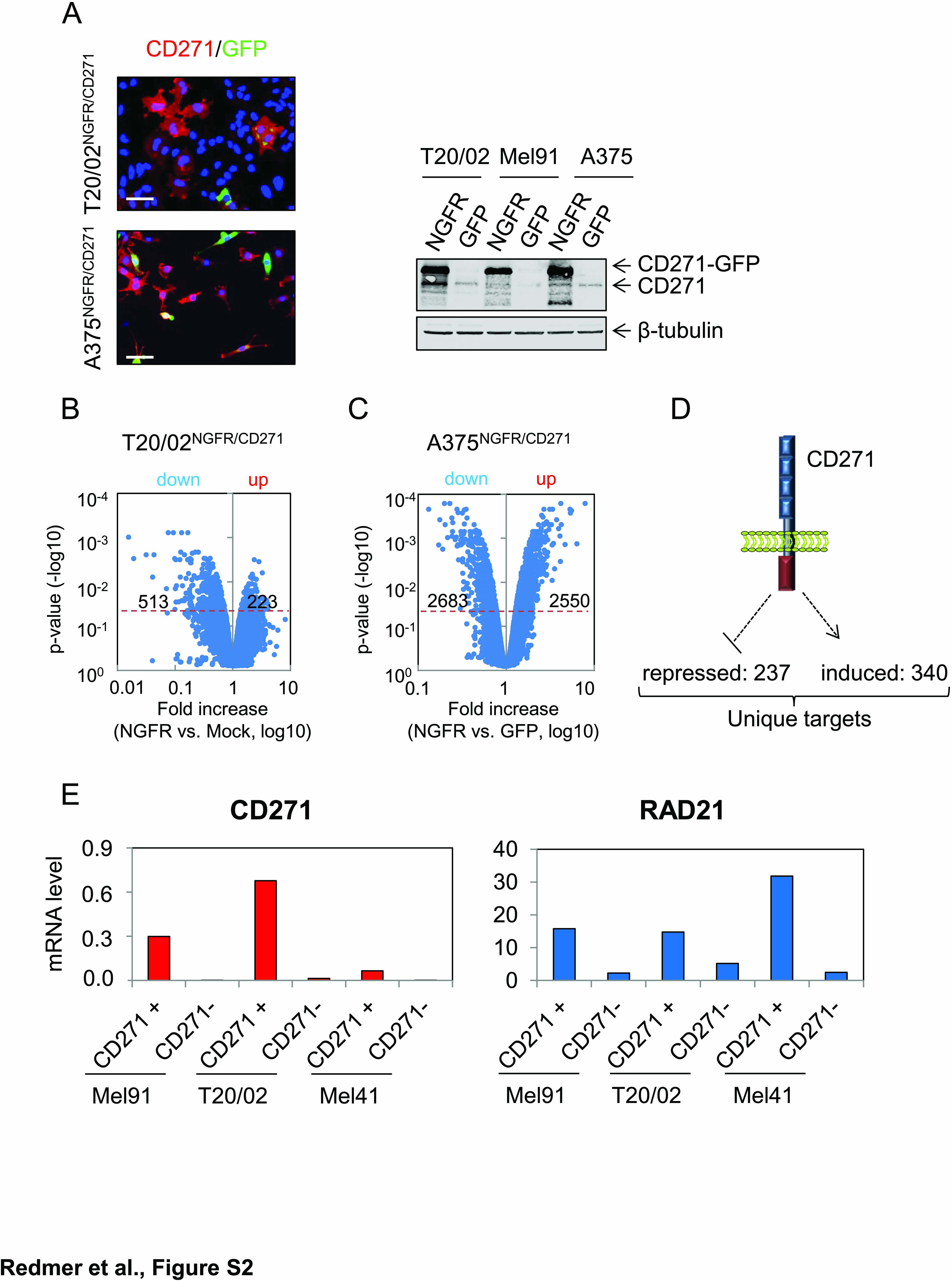

Supplement: Supplementary Figure 2 [file oncsis201688x9.tif]

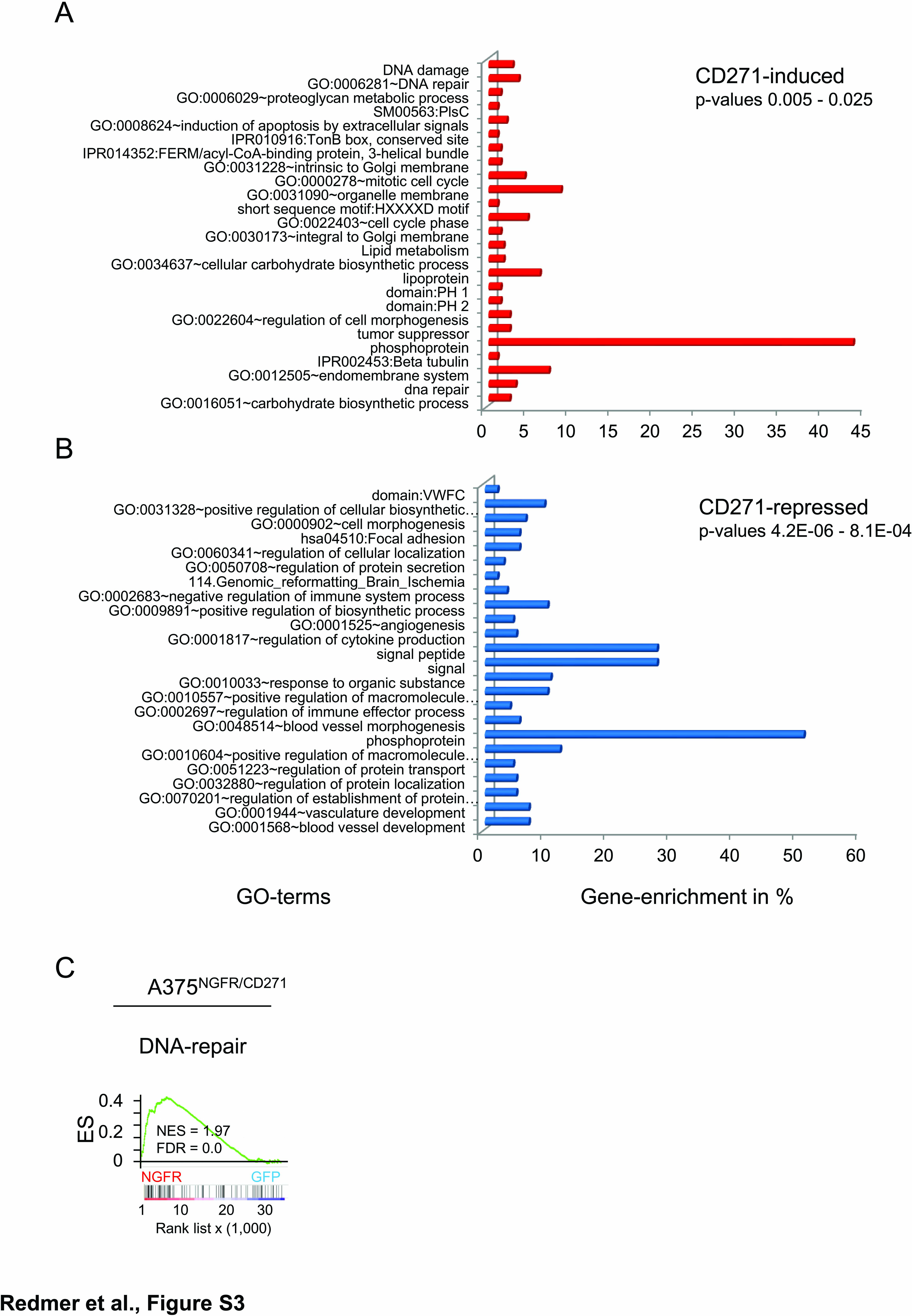

Supplement: Supplementary Figure 3 [file oncsis201688x10.tif]

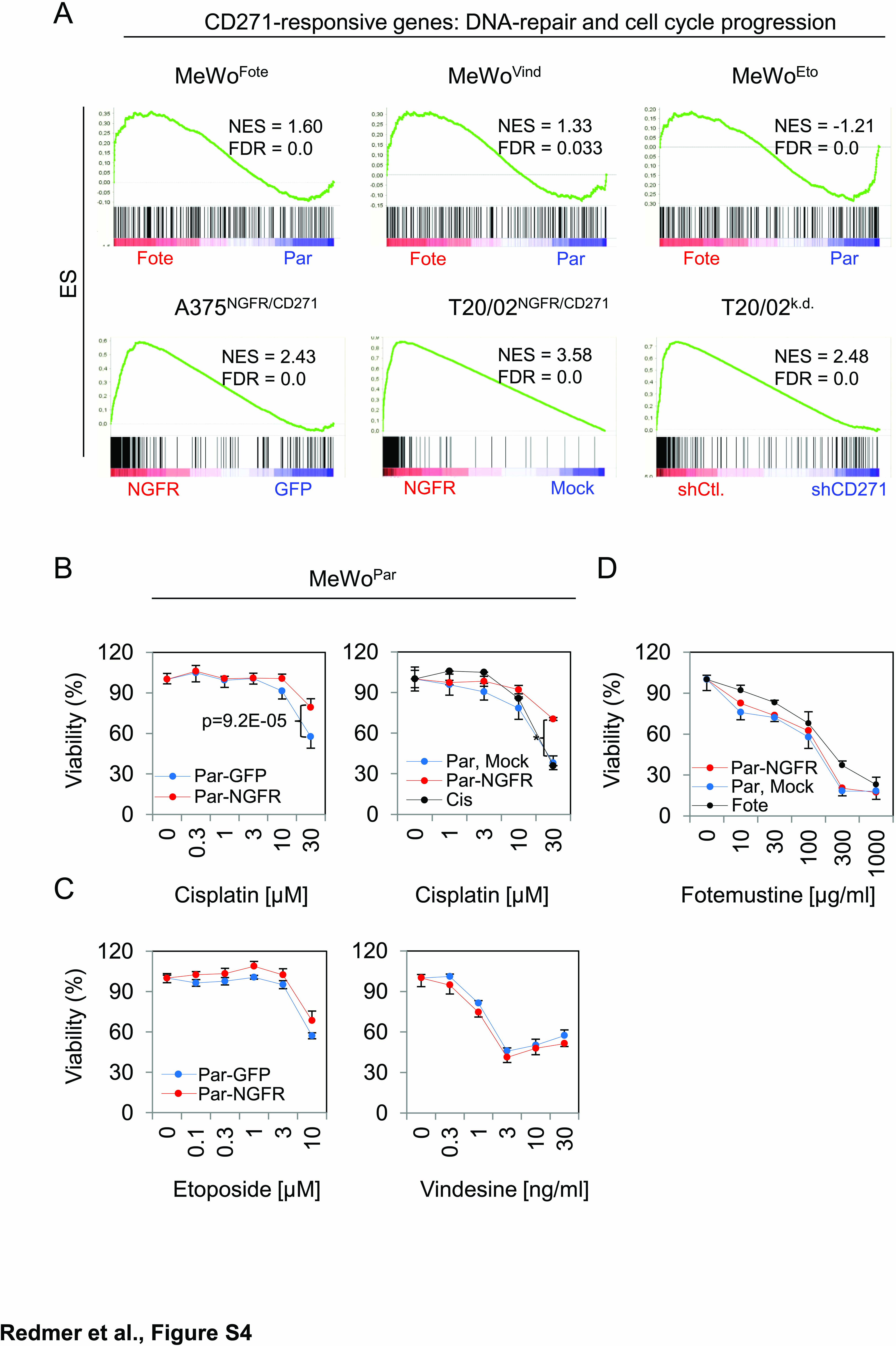

Supplement: Supplementary Figure 4 [file oncsis201688x11.tif]

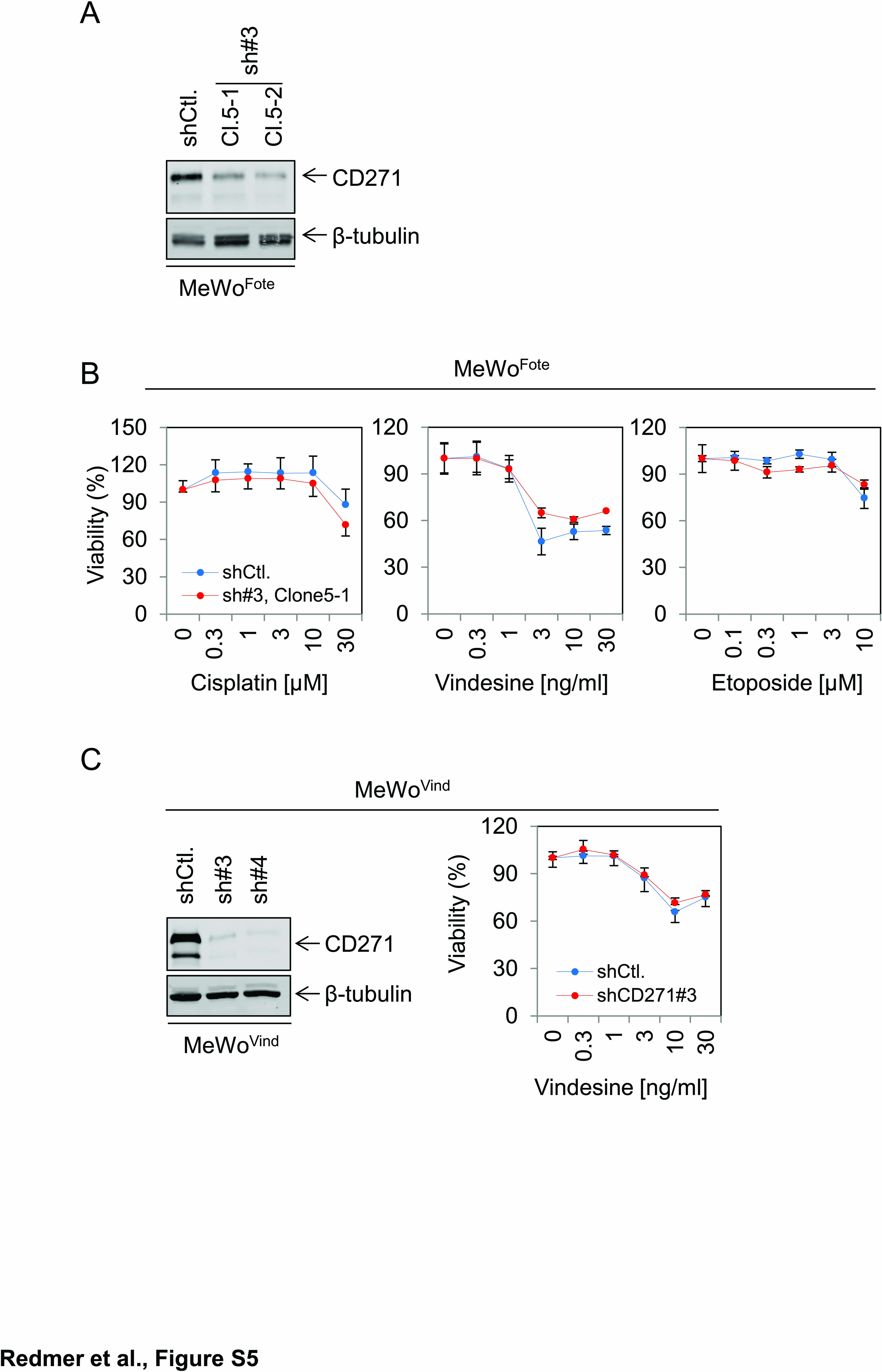

Supplement: Supplementary Figure 5 [file oncsis201688x12.tif]

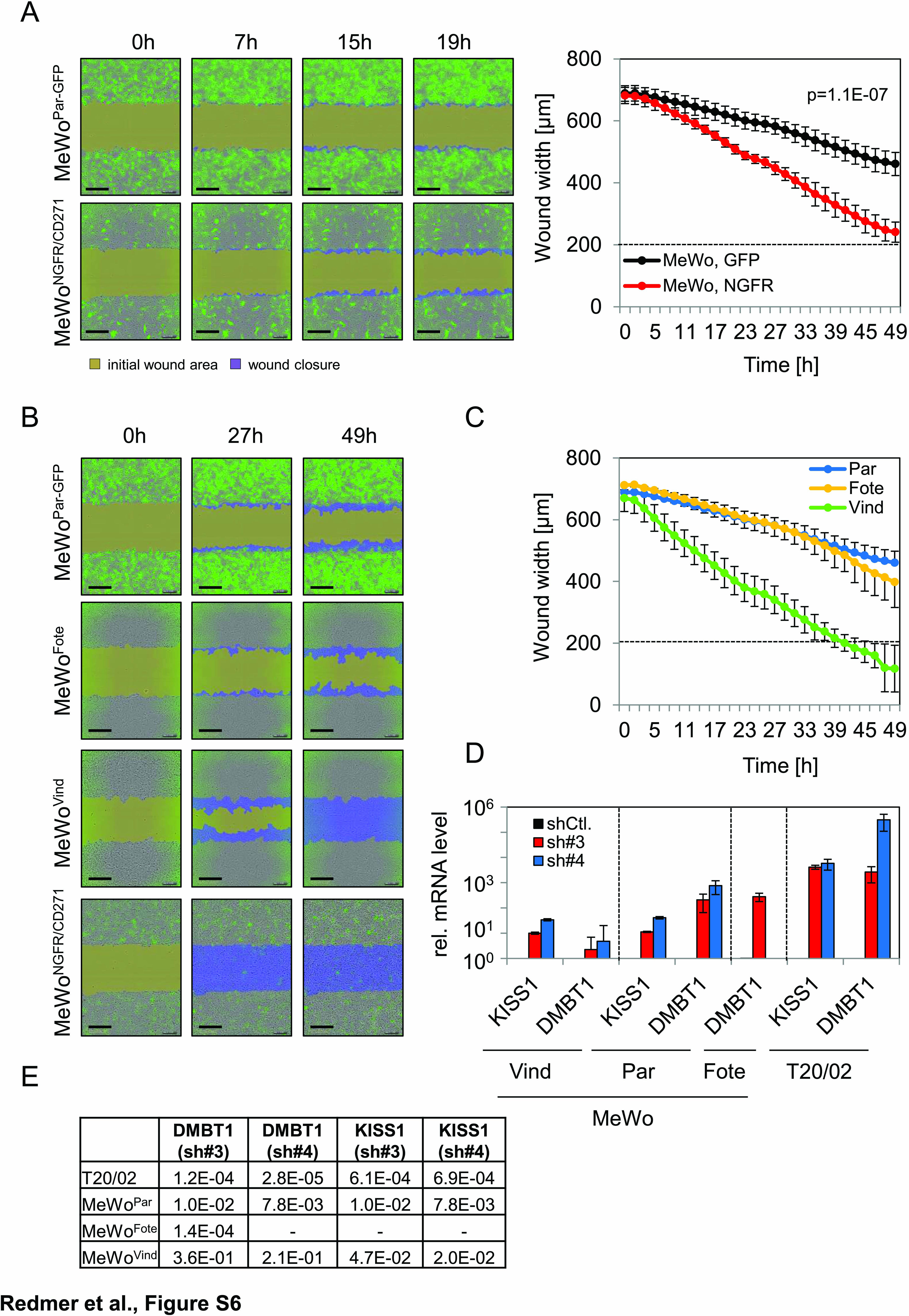

Supplement: Supplementary Figure 6 [file oncsis201688x13.tif]

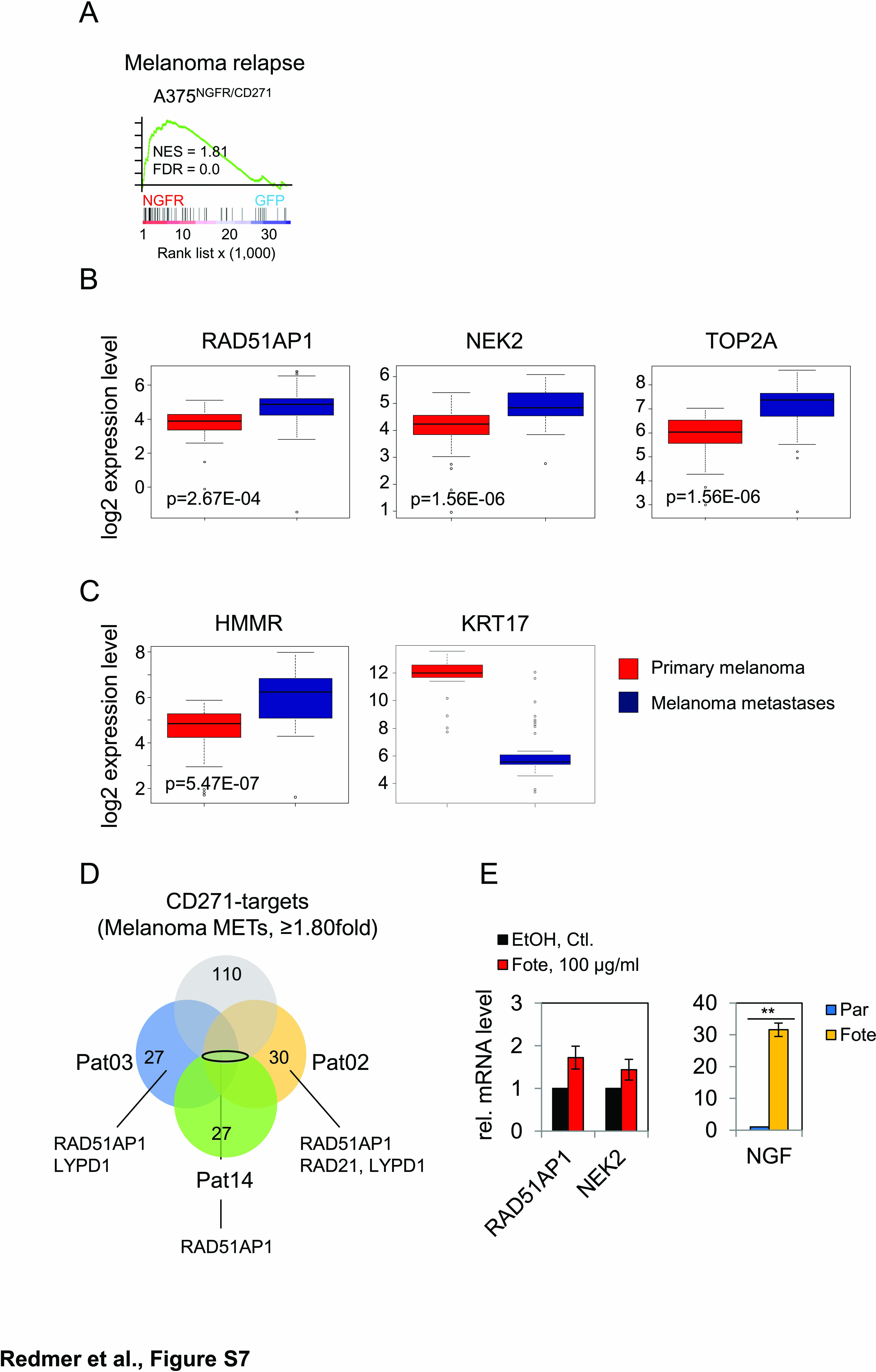

Supplement: Supplementary Figure 7 [file oncsis201688x14.tif]

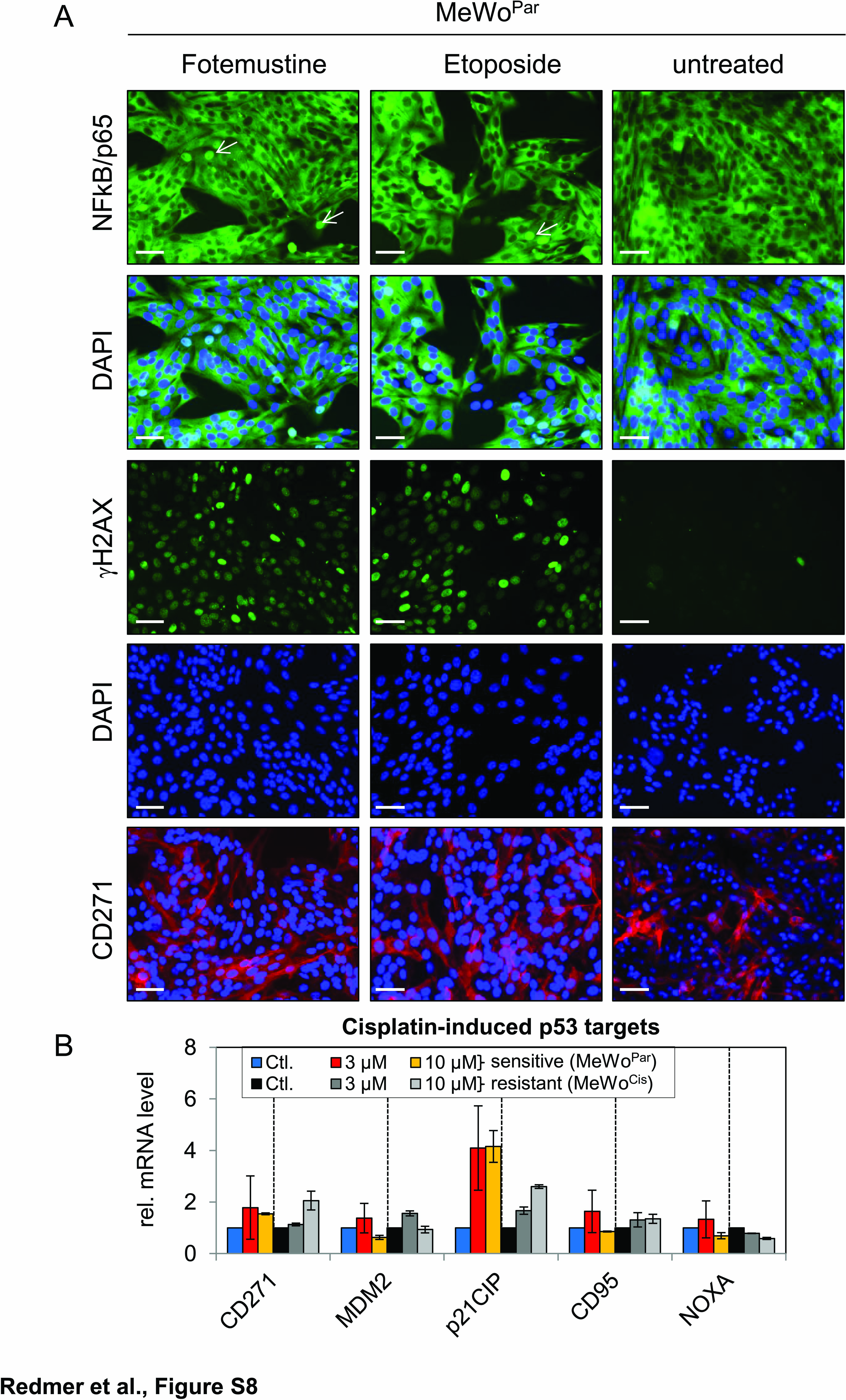

Supplement: Supplementary Figure 8 [file oncsis201688x15.tif]

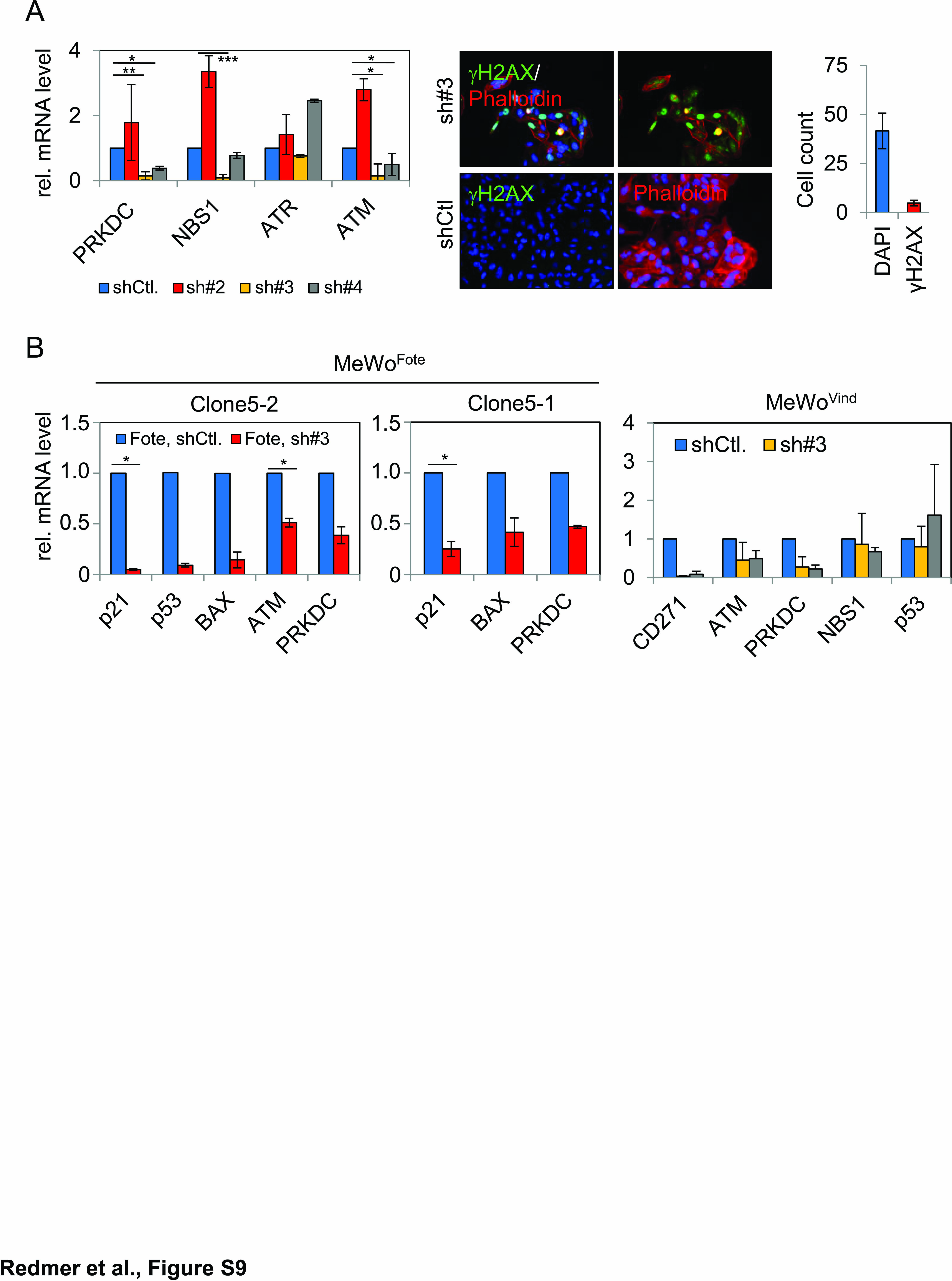

Supplement: Supplementary Figure 9 [file oncsis201688x16.tif]

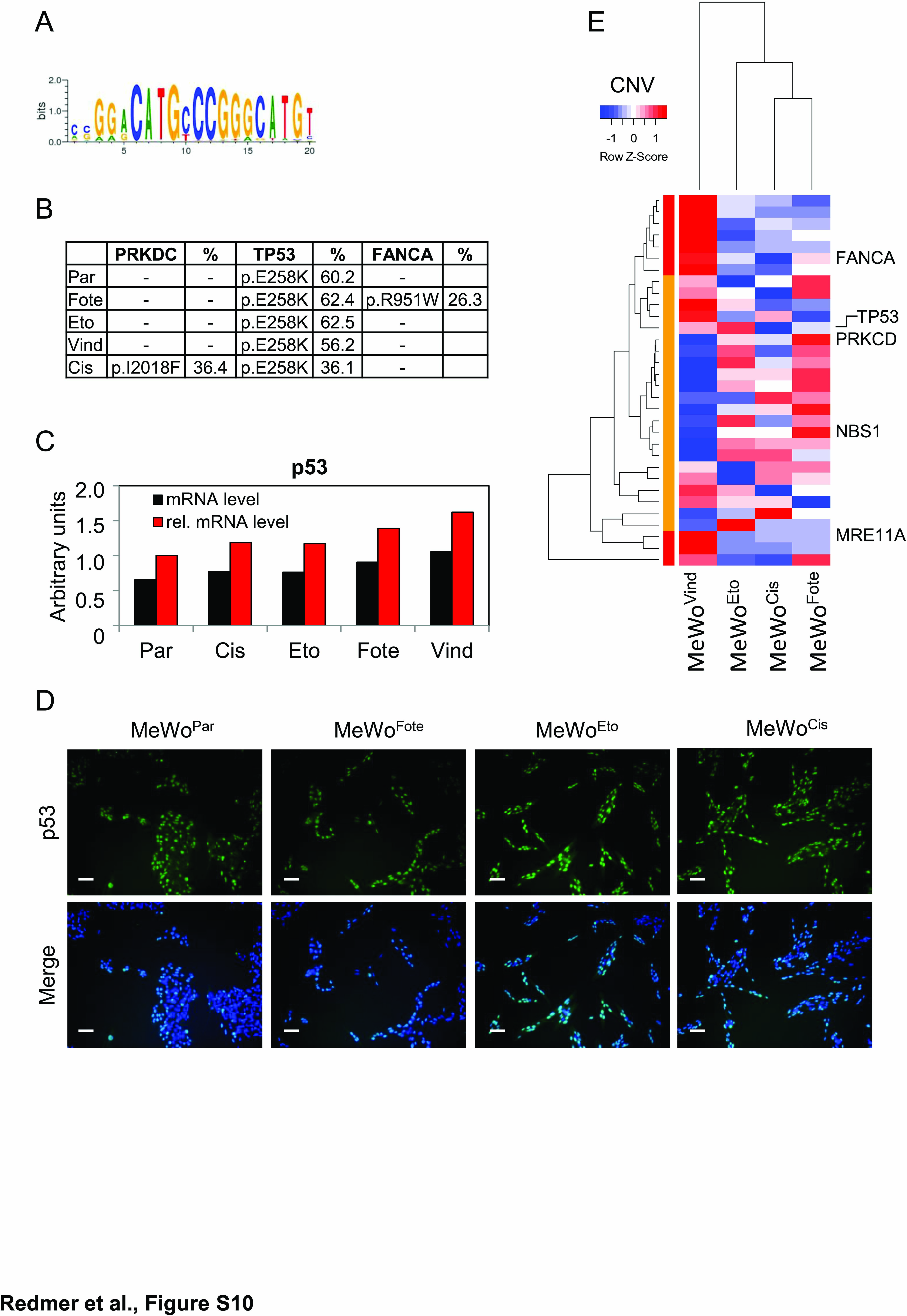

Supplement: Supplementary Figure 10 [file oncsis201688x17.tif]
